# Intra-Articular Platelet-Rich Plasma Injection After Anterior Cruciate Ligament Reconstruction: A Randomized Clinical Trial

**DOI:** 10.1001/jamanetworkopen.2024.10134

**Published:** 2024-05-10

**Authors:** Zipeng Ye, Huiang Chen, Yi Qiao, Chenliang Wu, Eunshinae Cho, Xiulin Wu, Ziyun Li, Jinlong Wu, Simin Lu, Guoming Xie, Shikui Dong, Junjie Xu, Jinzhong Zhao

**Affiliations:** 1 Department of Sports Medicine, Shanghai Sixth People’s Hospital Affiliated to Shanghai Jiao Tong University School of Medicine, Shanghai, China

## Abstract

**Importance:**

Platelet-rich plasma (PRP) has been considered a promising treatment for musculoskeletal disorders. The effects of PRP on clinical outcomes of anterior cruciate ligament reconstruction (ACLR) are controversial.

**Objective:**

To compare subjective outcomes and graft maturity in patients undergoing ACLR with and without postoperative intra-articular PRP injection.

**Design, Setting, and Participants:**

This surgeon- and investigator-masked randomized clinical trial included patients treated at a national medical center in China who were aged 16 to 45 years and scheduled to undergo ACLR. Participants were enrolled between March 21, 2021, and August 18, 2022, and followed up for 12 months, with the last participant completing follow-up on August 28, 2023.

**Interventions:**

Participants were randomized 1:1 to the PRP group (n = 60), which received 3 doses of postoperative intra-articular PRP injection at monthly intervals, or to the control group (n = 60), which did not receive postoperative PRP injection. Both groups had the same follow-up schedule.

**Main Outcomes and Measures:**

The primary outcome was the mean score for 4 subscales of the Knee Injury and Osteoarthritis Outcome Score (KOOS_4_) (range, 0-100, with higher scores indicating better knee function and fewer symptoms) at 12 months postoperatively. Secondary outcomes were patient-reported outcomes, graft maturity (on magnetic resonance imaging), and physical examinations at 3, 6, and 12 months.

**Results:**

Among the 120 randomized participants (mean [SD] age, 29.0 [8.0] years; 84 males [70%]), 114 (95%) were available for the primary outcome analysis. The mean KOOS_4_ scores at 12 months were 78.3 (SD, 12.0; 95% CI, 75.2-81.4) in the PRP group and 76.8 (SD, 11.9; 95% CI, 73.7-79.9) in the control group (adjusted mean between-group difference, 2.0; 95% CI, −2.3 to 6.3; *P* = .36). Secondary outcomes were not statistically significantly different between the 2 groups except for sports and recreation level and graft maturity at 6 months. Intervention-related adverse events included pain at the injection site and knee swelling after injection.

**Conclusions and Relevance:**

In this randomized clinical trial among patients undergoing ACLR, the addition of postoperative intra-articular PRP injection did not result in superior improvement of knee symptoms and function at 12 months compared with no postoperative injection. Further studies are required to determine appropriate indications for PRP in musculoskeletal disorders.

**Trial Registration:**

Chinese Clinical Trial Registry Identifier: ChiCTR2000040262

## Key Points

**Question:**

Does postoperative platelet-rich plasma (PRP) injection improve knee symptoms and function in patients undergoing anterior cruciate ligament reconstruction (ACLR)?

**Findings:**

In this randomized clinical trial of 120 participants undergoing ACLR, the intervention group received 3 doses of postoperative intra-articular PRP injection at monthly intervals, and the control group received no injection. The between-group difference in the Knee Injury and Osteoarthritis Outcome Score (mean score of 4 subscales) at 12 months was not statistically significant.

**Meaning:**

In this study among patients with ACLR, the addition of postoperative PRP injection did not significantly improve knee symptoms and function.

## Teaser

This randomized clinical trial compares knee symptoms and function in patients in China receiving and not receiving intra-articular platelet-rich plasma after anterior cruciate ligament (ACL) reconstruction.

## Introduction

The anterior cruciate ligament (ACL) is a primary stabilizer of the knee and vulnerable to sports injuries, which result in knee swelling, pain, and instability.^1,2^ ACL reconstruction (ACLR), one of the most common orthopedic surgeries, is performed in patients with a ruptured ACL to restore knee stability and facilitate a safe return to sports.^3,4^ During the first year postoperatively, knee symptoms and function gradually improve while the autograft or allograft undergoes a series of healing, remodeling, and maturation processes.^5,6^

Platelet-rich plasma (PRP) is a class of product derived from autologous blood through centrifugation, with high concentrations of growth factors and cytokines and potential for augmenting tissue repair procedures.^7,8^ It has been considered a promising therapy for musculoskeletal disorders, such as cartilage defects in knee osteoarthritis and tendinopathy in lateral epicondylitis, and for promoting tendon-to-bone healing after arthroscopic rotator cuff repair.^9,10,11,12^ Nevertheless, the clinical outcomes of PRP for ACLR are controversial^13,14,15,16,17^ due to the highly variable levels of evidence, methods of preparation, timing and doses of injection, and sites of administration. Recently, meta-analyses revealed that a single dose of intraoperative PRP injection (at graft harvest sites, in femoral and tibial tunnels, or into the joint cavity) was not associated with long-term clinical or radiologic improvements at more than 6 months postoperatively.^13,15^

Of note, intra-articular PRP injection has been expected to promote graft healing and remodeling,^9,13^ which typically occur during the months following ACLR.^5,18^ From this perspective, postoperative injections may have more real-time effects on biological transformation and functional recovery processes than conventional intraoperative administration.^19,20,21^ However, there is a lack of high-level comparative studies on the clinical efficacy and safety of postoperative PRP injection in patients with ACLR.

The purpose of this study was to compare the subjective outcomes and graft maturity in patients undergoing ACLR with and without postoperative intra-articular PRP injection. We hypothesized that the addition of postoperative intra-articular PRP injection would improve knee symptoms and function as well as graft maturity after ACLR.

## Methods

### Study Design

This surgeon- and investigator-masked randomized clinical trial (RCT) was performed at Shanghai Sixth People’s Hospital, a national medical center for orthopedics. The trial protocol and statistical analysis plan (Supplement 1) were approved by the institutional review board of the Shanghai Sixth People’s Hospital prior to participant enrollment on March 12, 2021. Participants signed written informed consent. This report follows the Consolidated Standards of Reporting Trials (CONSORT) reporting guideline.^22^

### Participants

Patients diagnosed with ACL injury by radiology or arthroscopy and scheduled to undergo ACLR were screened for eligibility. Eligible participants were aged 16 to 45 years with epiphyseal closure of the femur and tibia, and the affected knee had a nearly normal range of motion. Participant recruitment and baseline evaluation were completed during hospitalization within 3 days before surgery. Patients were excluded if they had multiple ligament injuries; bilateral ACL injuries; contralateral knee dysfunction; combined tibial plateau fracture; severe cartilage defect or osteoarthritis; concomitant meniscal repair; history of ipsilateral or contralateral knee surgery; blood system diseases (eg, low platelet count, coagulation disorders); or other systemic diseases or conditions that would affect postoperative treatment, rehabilitation, and follow-up assessments (detailed in the trial protocol in Supplement 1).

### Conventional Procedures

Participants underwent anatomic double-bundle ACLR by 1 of 3 fellowship-trained orthopedic surgeons (G.X., S.D., and J.Z.) specialized in arthroscopic knee surgery, following a standard operating procedure.^23,24^ The autologous semitendinosus tendon and gracilis tendon were harvested from the affected limb and braided to prepare two 4-stranded grafts. The tibial tunnel and femoral tunnel for the anteromedial bundle and posterolateral bundle were sequentially created using a transtibial method at 90° of knee flexion. The 4-stranded grafts were separately pulled into the femoral tunnels through the corresponding tibial tunnels, with the proximal ends suspended on cortical buttons. The fixation of the distal graft ends was implemented at full knee extension using a biocomposite interference screw and a cortical button with an adjustable loop setting through a transtibial ridge tunnel.

Participants received standardized rehabilitation education from a physical therapist through verbal instruction, printed brochures, and online videos before discharge (detailed training programs provided in the trial protocol in Supplement 1). The postoperative follow-up visits were scheduled at 4 weeks, 8 weeks, 3 months, 6 months, and 12 months.

### Group Allocation

To avoid imbalanced exclusion between groups, the randomization and group allocation were performed after surgery, and the demographic, radiologic, and surgical details were rechecked against the inclusion and exclusion criteria. Participants were randomized 1:1 to the PRP group or control group according to a computer-generated random number sequence. An independent trial assistant who was blocked from contacting the participants and assessors prepared the random numbers and sealed them in sequentially numbered opaque envelopes. At the first follow-up visit, the envelope with group allocation was revealed to each participant by an assistant who was not involved in the outcome measures.

To avoid low motivation for participation and potential ethical concerns because of nontherapeutic blood withdrawal in the control group (only for masking), participants were aware of their group allocation from the first follow-up visit, while the surgeons, physical therapist, clinical and radiologic outcome assessors (H.C., Y.Q., and E.C.), and statistician (J.X.) were masked to the group allocation throughout the study.

### Intervention

Participants in the PRP group received 3 doses of intra-articular PRP injection from a senior treatment nurse at 4 weeks (within 3 days), 8 weeks (within 5 days), and 3 months (within 7 days) postoperatively, referenced to graft remodeling processes and previous studies on knee osteoarthritis.^5,19,25^ At each follow-up visit, blood withdrawal and knee injection were performed after clinical assessments and radiologic scanning to avoid interference in outcome measures. Participants in the control group did not receive postoperative knee injection. Participants in both groups were instructed to avoid cointerventions (eg, unscheduled knee injection, nonsteroidal anti-inflammatory drugs) and to report additional medications or treatment to the clinician at follow-up visits.

The leukocyte-poor PRP was prepared using a 2-stage centrifugation method through a commercially used system (Platelet-Rich Plasma Preparation Kit; WEGO Ltd) as previously reported.^26,27^ In the first stage, 45 mL of venous blood was drawn and mixed with 5 mL of anticoagulant (acid-citrate-dextrose) preloaded in the syringe. The whole blood was centrifuged at 260*g* for 10 minutes and separated into 3 layers. In the second stage, the bottom layer (red blood cells) was discarded, and the upper and middle layers were aspirated and recentrifuged at 360*g* for 15 minutes. Then, the supernatant was discarded to obtain 5 mL of liquid-form leukocyte-poor PRP, of which the platelet concentration was 4.5 times that of the whole blood on average (range, 2.8-6.0 times) (detailed characteristics shown in eTable 1 in Supplement 2).

The intra-articular injection was performed immediately after PRP preparation with the participant in the sitting position at 90° of knee flexion, through an anterolateral approach, and without exogenous activation. To increase the accuracy of the knee injection, the puncture needle was located laterally to the patellar tendon and toward the intercondylar notch under ultrasound guidance.^28,29^ After injection, passive knee flexion and extension were performed for 10 cycles.

### Outcomes

The primary outcome was the mean score at 12 months for 4 subscales of the Knee Injury and Osteoarthritis Outcome Score (KOOS_4_).^30,31^ The KOOS has been validated for ACLR and includes 42 items covering 5 subscales: pain (9 items), symptoms (7 items), activities of daily living (17 items), sports and recreation (5 items), and quality of life (4 items).^32,33^ The responses are scored on a 5-point Likert scale (0-4), and scores for each subscale are standardized to range from 0 to 100, with higher scores indicating better knee-related results. The KOOS_4_ score excludes the subscale of activities of daily living to avoid the ceiling effect.^34^

Secondary outcomes were patient-reported outcomes (PROs), graft maturity, and physical examinations. The overall change of condition resulting from the treatment was assessed at 12 months using the Global Rating of Change (GROC) scale (range, −7 to 7, with higher positive values indicating more perceived improvement).^35^ Other PROs, including the KOOS subscales, 4-Item Pain Intensity Measure score (range, 0-40, with higher values indicating worse pain),^36^ Tegner score (range, 0 [sick leave or disability] to 10 [highest level of sports]),^37^ Lysholm score (range, 0-100, with higher scores indicating fewer knee-related symptoms),^38^ and subjective International Knee Documentation Committee (IKDC) score (range, 0-100, with higher scores indicating better performance in sports activities),^39^ were also assessed at 3, 6, and 12 months.

Participants underwent knee magnetic resonance imaging (MRI) (MAGNETOM Prisma; Siemens Healthineers) at the 3-, 6-, and 12-month follow-up visits. Graft maturity was evaluated by 2 independent investigators (H.C. and Y.Q.) on sagittal slices of the proton density–weighted sequence (repetition time, 3010 ms; echo time, 44 ms). The signal intensities were measured by placing unified 0.1-cm^2^ circular regions of interest on the background, quadriceps tendon, and ACL graft (separated into 6 segments).^40,41^ For each segment, the signal-to-noise quotient (SNQ) was calculated as (signal intensity of ACL graft − signal intensity of quadriceps tendon) / signal intensity of background. Lower SNQ values, representing reduced vascularity and water content, indicated better graft maturity.^42,43^

Physical examinations were performed by an experienced clinician (E.C.). The active assisted range of motion (knee flexion and extension) and knee circumference at the midpatellar level were measured at each follow-up visit.^44,45^ The knee laxity was evaluated using the anterior drawer, Lachman, and pivot-shift tests at 12 months (details provided in the trial protocol in Supplement 1).^46^

### Statistical Analysis

A priori sample size calculation was performed using G*Power, version 3.1 software (Heinrich-Heine-Universität Düsseldorf). To detect a 13-point, anchor-based minimal clinically important difference (MCID) of the KOOS_4_ score between the 2 groups with a common SD of 20,^30,31,47,48^ a total of 120 participants (60 in each group) was required for a 2-sided *P* < .05, a power of 90%, and an allowed dropout rate of 15%.

The primary analysis was performed according to the intention-to-treat principle. To compare the primary outcome between the 2 groups, an analysis of covariance model was applied with adjustment for the baseline KOOS_4_.^30,31,49^ Other PROs during follow-up visits (except for GROC scale) were also compared using analysis of covariance models adjusting for the corresponding baseline scores. In addition, the Mann-Whitney-Wilcoxon test and Student *t* test were used to compare ordinal or nonnormally distributed outcomes (eg, GROC scale, graft SNQ, or knee laxity) and normally distributed outcomes, respectively. The Pearson χ^2^ test or Fisher exact test was used to compare dichotomous outcomes. The interrater reliability of graft maturity was evaluated using the intraclass correlation coefficient and classified as good (≥0.75), fair (0.50-0.74), and poor (<0.50).

Sensitivity analyses included missing data, multivariable, and per-protocol analyses. For outcomes with missing data of more than 5%, multiple imputation was planned using the fully conditional specifications method; variables in the imputation model included group allocation, age, sex, sports participation, graft diameter, meniscal treatment, and baseline KOOS_4_.^50^ These potential outcome variables and confounders were also included in the multivariable linear regression model for the primary outcome. The per-protocol (as-treated) analysis excluded participants receiving fewer than 3 doses of PRP injection in the PRP group and participants undergoing reoperation or unscheduled knee injection in both groups.

All statistical analyses were performed using SPSS, version 27.0 software (IBM Corporation), with a 2-sided *P* < .05. Because of the potential for type I error due to multiple comparisons, analyses of secondary outcomes were exploratory.

## Results

### Participants

**Figure 1.**
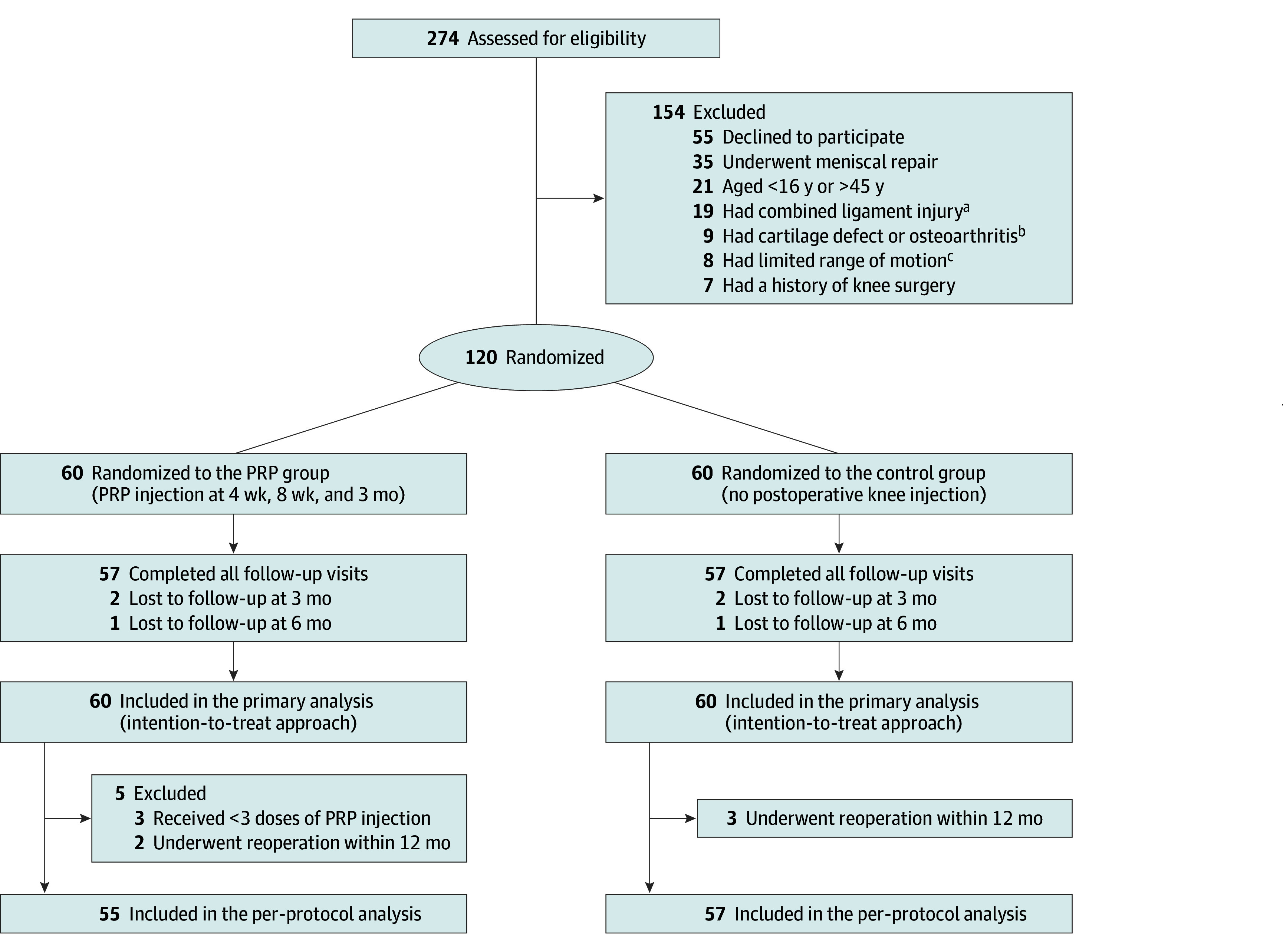
Flowchart of Participant Enrollment, Allocation, Follow-Up, and Analysis PRP indicates platelet-rich plasma. ^a^Restricted knee flexion (<120°) or restricted knee extension (>5°), measured by placing the axis of a goniometer over the lateral femoral epicondyle. ^b^Including posterior cruciate ligament injury, posterolateral complex injury, medial patellofemoral ligament injury, and grade 2 or 3 medial collateral ligament injury. ^c^Grade 3 or 4 (cartilage defects extending down >50% of cartilage depth) of the International Cartilage Repair Society grading, evaluated by arthroscopy during surgery.

Enrollment took place between March 21, 2021, and August 18, 2022, and the last participant completed the 12-month follow-up visit on August 28, 2023. Of the 274 patients screened for eligibility, 99 were ineligible, and 55 declined to participate (Figure 1). A total of 120 participants (mean [SD] age, 29.0 [8.0] years; 36 females [30%]; 84 males [70%]) were thus randomized 1:1 and included in the primary analysis. The baseline characteristics were comparable between the PRP and control groups (Table 1).

**Table 1.** Baseline Characteristics of Study Participants

Characteristic	No. of participants (%)	
	PRP group (n = 60)	Control group (n = 60)
Age, mean (SD), y	28.0 (7.9)	30.0 (8.0)
Sex		
Female	17 (28.3)	19 (31.7)
Male	43 (71.7)	41 (68.3)
BMI, mean (SD)	24.8 (3.7)	25.2 (3.6)
Affected side		
Left	23 (38.3)	29 (48.3)
Right	37 (61.7)	31 (51.7)
Beighton score, median (IQR)^a^	2 (0-3)	1 (0-4)
Preinjury Tegner score, mean (SD)^b^	7.0 (1.6)	7.1 (1.9)
Time from injury to surgery, median (IQR), mo	3 (1-9)	3 (1-8)
Sports participation at injury		
Contact pivoting	34 (56.7)	32 (53.3)
Noncontact pivoting	20 (33.3)	18 (30.0)
Nonpivoting	6 (10.0)	10 (16.7)
Preoperative range of motion, mean (SD), degrees^c^		
At knee flexion	132.3 (6.7)	131.3 (7.8)
At knee extension	−1.8 (2.8)	−2.0 (2.8)
Knee laxity under anesthesia^d^		
Anteroposterior laxity by Lachman test		
Grade 1	7 (11.7)	6 (10.0)
Grade 2	36 (60.0)	38 (63.3)
Grade 3	17 (28.3)	16 (26.7)
Rotatory laxity by pivot-shift test		
Grade 1	25 (41.7)	27 (45.0)
Grade 2	24 (40.0)	25 (41.7)
Grade 3	11 (18.3)	8 (13.3)
Amount of ACL remnant by arthroscopy^e^		
Grade 0	33 (55.0)	27 (45.0)
Grade 1	14 (23.3)	14 (23.3)
Grade 2	5 (8.3)	7 (11.7)
Grade 3	8 (13.3)	12 (20.0)
Graft diameter of AMB, mean (SD), mm^f^	7.8 (0.7)	7.8 (0.6)
Graft diameter of PLB, mean (SD), mm^f^	5.9 (0.5)	5.9 (0.5)
Meniscal treatment at surgery		
None	42 (70.0)	44 (73.3)
Partial medial meniscectomy	8 (13.3)	7 (11.7)
Partial lateral meniscectomy	15 (25.0)	11 (18.3)
Baseline KOOS, mean (SD)^g^		
KOOS_4_	52.9 (16.6)	55.1 (16.0)
KOOS pain	79.6 (14.8)	82.0 (15.7)
KOOS symptoms	62.0 (21.5)	65.0 (17.7)
KOOS activities of daily living	86.9 (15.6)	84.7 (17.5)
KOOS sports and recreation	41.0 (26.8)	43.0 (25.7)
KOOS quality of life	29.1 (20.6)	30.2 (23.7)
Baseline P_4_ score by VAS, mean (SD)^h^	8.0 (5.1)	7.4 (5.2)
Baseline Lysholm score, mean (SD)^i^	72.1 (18.5)	72.3 (20.3)
Baseline subjective IKDC score, mean (SD)^j^	62.0 (18.0)	60.5 (18.4)

Abbreviations: ACL, anterior cruciate ligament; AMB, anteromedial bundle; BMI, body mass index (as measured by weight in kilograms divided by height in meters squared); IKDC, International Knee Documentation Committee; KOOS, Knee Injury and Osteoarthritis Outcome Score; P_4_, 4-Item Pain Intensity Measure; PLB, posterolateral bundle; PRP, platelet-rich plasma; VAS, visual analog scale.

^a^ Beighton score includes 5 maneuvers to test the hyperextension of multiple joints; scores range from 0 to 9, with higher scores indicating greater extent of joint hypermobility.

^b^ The Tegner Activity Scale assesses the highest level of current sports participation; scores range from 0 (sick leave or disability) to 10 (professional level of competitive sports).

^c^ The range of motion is measured by placing the axis of a goniometer over the lateral femoral epicondyle and aligning the 2 arms along the femoral and fibular axes, with positive values indicating knee flexion and negative values indicating hyperextension.

^d^ The Lachman test represents knee anteroposterior laxity at 30° of flexion; grades include 0 (0-2 mm), 1 (3-5 mm), 2 (6-10 mm), and 3 (>10 mm) based on the side-to-side difference. The pivot-shift test is graded as 0 (negative), 1 (glide), 2 (clunk), and 3 (gross reduction), with higher grades indicating increased knee rotatory laxity.

^e^ The amount of ACL remnant is graded as 0 (no remnant), 1 (less than one-third), 2 (one- to two-thirds), and 3 (greater than two-thirds) based on the length proportion covering the reconstructed ACL.

^f^ The graft sizes of AMB and PLB are measured by passing the 4-stranded semitendinosus tendon and gracilis tendon, respectively, through a nonslotted diameter measurement tool.

^g^ The KOOS includes 42 items covering 5 subscales (pain, symptoms, activities of daily living, sports and recreation, and quality of life). Scores for each subscale range from 0 to 100, with higher scores indicating better knee-related results. The KOOS_4_ is calculated as the mean score for 4 of the 5 subscales (except for activities of daily living).

^h^ The VAS for pain is assessed on a straight horizontal line, with scores ranging from 0 (no pain) to 10 (worst pain possible). The P_4_ score is calculated as the sum of VAS scores at 4 time points (morning, afternoon, evening, and with activity) during the past 2 days.

^i^ The Lysholm Knee Questionnaire includes 8 items on subjective perception (eg, instability, pain, or locking) related to knee ligament injury; scores range from 0 to 100, with higher scores indicating fewer symptoms and better function in daily living.

^j^ The IKDC Subjective Knee Form includes 18 items covering 3 domains (symptoms, sports activities, and function); scores range from 0 (worst condition) to 100 (best condition).

At 12 months, 114 participants (95%) were available for analysis of the primary and secondary outcomes, and 6 participants (3 in the PRP group and 3 in the control group) were lost to follow-up (5% missing data). Three participants in the PRP group who received fewer than 3 doses of PRP injection and 5 participants (2 in the PRP group and 3 in the control group) who underwent reoperation within 12 months were excluded from the per-protocol analysis.

### Primary Outcome

**Figure 2.**
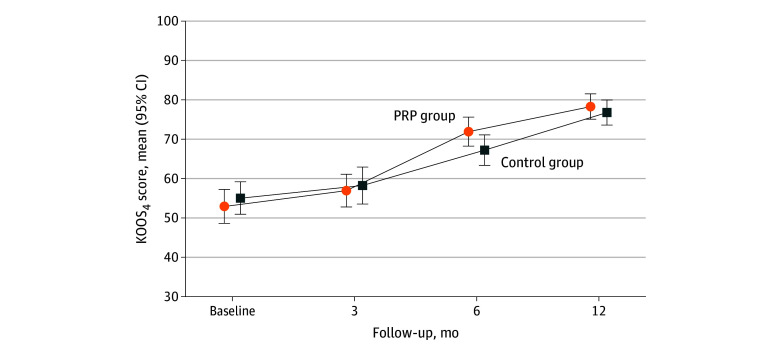
Mean Scores of the 4 Subscales of the Knee Injury and Osteoarthritis Outcome Score (KOOS_4_) at Baseline and During 12-Month Follow-Up by Group The KOOS_4_ is calculated as the mean score for the pain, symptoms, sports and recreation, and quality-of-life subscales of the KOOS. Scores range from 0 to 100, with higher scores indicating better knee-related results. The means and 95% CIs are unadjusted. The between-group differences at follow-up visits were adjusted for the baseline KOOS_4_ using an analysis of covariance model, with positive values indicating higher scores in the platelet-rich plasma (PRP) group and negative values indicating higher scores in the control group (3 months: mean, −0.4 [95% CI, −6.3 to 5.6]; 6 months: mean, 5.5 [95% CI, 0.4-10.6]; 12 months: mean, 2.0 [95% CI, −2.3 to 6.3]).

The mean KOOS_4_ scores at 12 months were 78.3 (SD, 12.0; 95% CI, 75.2-81.4) in the PRP group and 76.8 (SD, 11.9; 95% CI, 73.7-79.9) in the control group (Figure 2); no statistically significant difference was found between groups (adjusted mean difference, 2.0; 95% CI, –2.3 to 6.3; *P* = .36). The missing data, multivariable, and per-protocol analyses did not yield different results (eTables 2 and 3 in Supplement 2).

### Secondary Outcomes

The GROC scale and other PROs assessed at 3 and 12 months were not statistically significantly different between groups (Table 2). The PRP group showed more favorable results than the control group in 2 PROs assessed at 6 months: the KOOS subscale of sports and recreation (adjusted mean difference, 6.8; 95% CI, 0.1 to 13.6; *P* = .046) and the Tegner score (adjusted mean difference, 0.8; 95% CI, 0.3 to 1.3; *P* = .001).

**Table 2.** Patient-Reported Outcomes During Follow-Up Visits

Outcome	Mean (SD)		Adjusted between-group difference, mean (95% CI)^a^	*P* value
	PRP group	Control group		
**Primary outcome**				
No. of participants	57	57	NA	NA
KOOS_4_ at 12 mo^b^	78.3 (12.0)	76.8 (11.9)	2.0 (−2.3 to 6.3)	.36
**Secondary outcomes**				
GROC at 12 mo, median (IQR)^c^	5 (4 to 6)	5 (3 to 5)	NA	.29
PROs at 3 mo				
No. of participants	59	58	NA	NA
KOOS pain	77.4 (14.1)	78.7 (16.1)	−0.5 (−5.9 to 4.9)	.85
KOOS symptoms	60.6 (17.2)	60.8 (19.0)	0.7 (−5.7 to 7.2)	.83
KOOS activities of daily living	87.6 (11.7)	87.7 (11.8)	−0.2 (−4.4 to 4.1)	.94
KOOS sports and recreation	48.3 (26.1)	49.1 (26.0)	0.3 (−8.8 to 9.5)	.94
KOOS quality of life	41.4 (20.3)	44.4 (22.0)	−2.6 (−9.6 to 4.5)	.47
P_4_ score by VAS^d^	6.4 (5.3)	6.2 (4.7)	0 (−1.8 to 1.7)	.96
Tegner score^e^	2.5 (1.1)	2.4 (1.3)	0.1 (−0.3 to 0.6)	.58
Lysholm score^f^	71.0 (17.3)	72.9 (17.2)	−1.7 (−7.8 to 4.4)	.58
Subjective IKDC score^g^	60.2 (14.0)	60.5 (15.4)	−0.4 (−5.7 to 4.9)	.88
PROs at 6 mo				
No. of participants	59	57	NA	NA
KOOS pain	86.3 (10.6)	83.6 (10.5)	3.4 (−0.2 to 7.1)	.07
KOOS symptoms	72.6 (17.9)	68.4 (18.8)	5.1 (−1.4 to 11.6)	.13
KOOS activities of daily living	94.8 (6.6)	92.3 (9.0)	2.4 (−0.5 to 5.2)	.10
KOOS sports and recreation	72.9 (18.9)	66.9 (19.1)	6.8 (0.1 to 13.6)	.046
KOOS quality of life	56.0 (20.5)	50.1 (21.1)	6.3 (−1.2 to 13.7)	.10
P_4_ score by VAS^d^	2.8 (2.7)	3.1 (3.3)	−0.4 (−1.5 to 0.7)	.48
Tegner score^e^	4.4 (1.6)	3.7 (1.3)	0.8 (0.3 to 1.3)	.001
Lysholm score^f^	82.4 (13.4)	78.7 (15.6)	4.0 (−1.1 to 9.1)	.13
Subjective IKDC score^g^	74.6 (11.3)	72.5 (11.6)	2.0 (−2.0 to 6.0)	.32
PROs at 12 mo				
No. of participants	57	57	NA	NA
KOOS pain	88.9 (9.0)	87.6 (9.1)	1.8 (−1.4 to 5.0)	.26
KOOS symptoms	76.5 (13.5)	77.4 (14.8)	−0.5 (−5.5 to 4.6)	.86
KOOS activities of daily living	96.7 (4.7)	94.9 (7.0)	1.6 (−0.6 to 3.8)	.15
KOOS sports and recreation	81.1 (16.1)	79.8 (15.4)	1.6 (−4.1 to 7.4)	.58
KOOS quality of life	66.8 (19.9)	62.4 (19.1)	4.7 (−2.4 to 11.8)	.19
P_4_ score by VAS^d^	1.8 (2.2)	2.0 (2.7)	−0.2 (−1.1 to 0.7)	.70
Tegner score^e^	5.5 (1.8)	5.3 (1.6)	0.3 (−0.2 to 0.8)	.24
Lysholm score^f^	83.9 (12.0)	84.7 (11.7)	−0.7 (−5.0 to 3.7)	.76
Subjective IKDC score^g^	80.9 (12.0)	79.9 (9.6)	0.7 (−3.1 to 4.5)	.71

Abbreviations: GROC, Global Rating of Change; IKDC, International Knee Documentation Committee; KOOS, Knee Injury and Osteoarthritis Outcome Score; NA, not applicable; P_4_, 4-Item Pain Intensity Measure; PRO, patient-reported outcome; PRP, platelet-rich plasma; VAS, visual analog scale.

^a^ The mean between-group difference for each subjective outcome (except for GROC scale) is adjusted for the corresponding baseline (preoperative) score using an analysis of covariance model, with positive values indicating higher scores in the PRP group and negative values indicating higher scores in the control group.

^b^ The KOOS includes 42 items covering 5 subscales (pain, symptoms, activities of daily living, sports and recreation, and quality of life). Scores for each subscale range from 0 to 100, with higher scores indicating better knee-related results. The KOOS_4_ is calculated as the average score for 4 of the 5 subscales (except for activities of daily living).

^c^ The GROC scale assesses the overall change of condition resulting from the treatment (surgery and postoperative treatment). Scores range from −7 (a very great deal worse) to 7 (a very great deal better), with higher positive values indicating more improvement.

^d^ The VAS for pain is assessed on a straight horizontal line, with scores ranging from 0 (no pain) to 10 (worst pain possible). The P_4_ score is calculated as the sum of VAS scores at 4 time points (morning, afternoon, evening, and with activity) during the past 2 days.

^e^ The Tegner Activity Scale assesses the highest level of current sports participation; scores range from 0 (sick leave or disability) to 10 (professional level of competitive sports).

^f^ The Lysholm Knee Questionnaire includes 8 items regarding subjective perception (eg, instability, pain, or locking) related to knee ligament injury; scores range from 0 to 100, with higher scores indicating fewer symptoms and better function in daily living.

^g^ The IKDC Subjective Knee Form includes 18 items covering 3 domains (symptoms, sports activities, and function); scores range from 0 (worst condition) to 100 (best condition).

The interrater reliability of graft SNQ values was good (intraclass correlation coefficient, 0.88). The PRP group demonstrated improved graft maturity at 6 months in the intra-articular and femoral intratunnel segments of both the anteromedial bundle (median, 10.3 [IQR, 5.9-13.8; *P* = .03] and 13.4 [IQR, 10.2-17.7; *P* = .003], respectively) and the posterolateral bundle (median, 11.4 [IQR, 7.1-17.4; *P* = .007] and 15.4 [IQR, 10.9-21.0; *P* = .02], respectively) compared with the control group (Table 3). No statistically significant between-group differences were found for graft maturity on the 3- and 12-month follow-up MRI. There were no statistically significant between-group differences in the results of follow-up physical examinations (eTable 4 in Supplement 2). These results were similar in the per-protocol analysis (eTables 5-7 in Supplement 2).

**Table 3.** Graft Maturity on Magnetic Resonance Imaging During Follow-Up Visits

Outcome	Median (IQR)		*P* value
	PRP group	Control group	
**Graft SNQ at 3 mo** ^a^			
No. of participants	59	58	NA
Anteromedial bundle			
Femoral intratunnel segment^b^	9.2 (5.7-15.6)	11.5 (4.2-16.6)	.95
Intra-articular segment	6.6 (4.2-11.0)	6.2 (3.4-10.2)	.54
Tibial intratunnel segment	6.0 (2.3-9.7)	4.4 (2.1-8.0)	.26
Posterolateral bundle			
Femoral intratunnel segment	9.3 (4.4-14.5)	11.3 (5.2-17.3)	.40
Intra-articular segment	8.4 (4.8-12.9)	7.5 (3.8-12.6)	.48
Tibial intratunnel segment	6.0 (3.4-9.8)	5.1 (2.7-8.3)	.41
**Graft SNQ at 6 mo**			
No. of participants	59	57	NA
Anteromedial bundle			
Femoral intratunnel segment	13.4 (10.2-17.7)	17.4 (12.8-21.5)	.003
Intra-articular segment	10.3 (5.9-13.8)	13.6 (8.7-17.6)	.03
Tibial intratunnel segment	10.2 (6.4-13.6)	9.5 (5.7-14.7)	.90
Posterolateral bundle			
Femoral intratunnel segment	15.4 (10.9-21.0)	19.6 (14.2-24.1)	.02
Intra-articular segment	11.4 (7.1-17.4)	15.1 (11.2-19.5)	.007
Tibial intratunnel segment	11.1 (6.1-16.6)	10.1 (5.9-16.5)	.81
**Graft SNQ at 12 mo**			
No. of participants	57	57	NA
Anteromedial bundle			
Femoral intratunnel segment	9.8 (6.1-17.4)	12.3 (8.1-17.7)	.12
Intra-articular segment	8.1 (5.6-11.4)	9.5 (5.1-13.6)	.38
Tibial intratunnel segment	8.7 (5.9-11.4)	8.1 (5.0-11.7)	.63
Posterolateral bundle			
Femoral intratunnel segment	10.4 (6.2-19.9)	13.8 (8.8-20.3)	.19
Intra-articular segment	7.9 (5.5-11.8)	10.2 (5.8-14.6)	.16
Tibial intratunnel segment	7.6 (5.3-10.9)	7.2 (4.0-11.4)	.94

Abbreviations: ACL, anterior cruciate ligament; NA, not applicable; PRP, platelet-rich plasma; SNQ, signal-to-noise quotient.

^a^ The graft maturity is evaluated on the sagittal plane of fat-saturated proton density–weighted magnetic resonance imaging. The SNQ is calculated as (signal intensity of ACL graft – signal intensity of quadriceps tendon) / signal intensity of background, with lower SNQ values indicating better graft maturity. The quadriceps tendon and background are located at the patellar upper limit level and at 2 cm anterior to the patellar tendon, respectively.

^b^ The SNQ values are separately calculated for 6 segments of the reconstructed ACL as follows: the femoral intratunnel segment, intra-articular segment, and tibial intratunnel segment of the anteromedial bundle and posterolateral bundle. In each segment, 3 regions of interest (0.1-cm^2^ circles) are selected, and the mean signal intensity is calculated.

### Adverse Events

The intervention-related adverse events in the PRP group were mild and temporary, with 4 participants (6.7%) reporting pain at the injection site and 3 (5.0%) reporting knee swelling after injection. These symptoms were relieved within 5 days, and no additional medications were required. The rates of common adverse events and additional medications were comparable between groups (eTable 8 in Supplement 2).

## Discussion

The most important finding of this study was that 3-dose postoperative intra-articular PRP injection compared with no injection did not significantly improve knee symptoms and function at 12 months after ACLR. The adjusted between-group difference in the primary outcome was not statistically significant, and the upper limit of the 95% CI of the mean difference did not achieve the MCID (ranging from 7.9 to 18.3),^47,48,51^ excluding the clinical significance of this treatment.

Most secondary outcomes were not statistically significantly different between groups except for sports and recreation level and graft maturity at 6 months. The between-group differences in the KOOS subscale of sports and recreation and Tegner score were statistically significant (favoring the PRP group); the mean differences did not achieve the MCIDs, while the respective upper limits of the 95% CIs slightly exceeded the MCIDs (12.5 for KOOS sports and recreation; 1 for Tegner score).^48^ Nevertheless, the results of the secondary outcomes were inconclusive, with variable levels of type I and type II errors because the significance level and a priori sample size calculations were based on the primary outcome.

The clinical significance of statistically improved graft maturity at 6 months in the PRP group remains ambiguous. Magnetic resonance imaging has been a widely used, noninvasive method to evaluate graft revascularization and maturation after ACLR.^40,41,42,43^ Although graft maturity can be compared using the SNQ value, no studies have reported the MCID or grading system for this measure. Furthermore, the association between graft maturity and PROs or knee stability has not been fully elucidated despite shared risk factors for poor outcomes.^40,52,53,54^ Therefore, further investigations are warranted to better understand the clinical relevance of graft maturity (perhaps guiding an accelerated rehabilitation program for patients with superior graft maturity or a delayed return to sports for patients with inferior graft maturity).^55^

Recently, several systematic reviews of RCTs reached a consensus that intraoperative injection of PRP is ineffective in improving long-term PROs, knee stability, or radiologic outcomes at more than 6 months after ACLR despite different results for short-term outcomes.^13,14,15,16^ Similarly, the 12-month clinical and radiologic outcomes of the present study showed no long-term efficacy of postoperative PRP injection in patients who underwent ACLR. A meta-analysis^13^ stratified the PROs by follow-up timing and found that intraoperative PRP injection was favorable only in terms of visual analog scale IKDC scores at 3 months. Another meta-analysis^15^ with pooled follow-up periods showed improved Lysholm and visual analog scale scores in the PRP group; the between-group differences were statistically but not clinically significant. Furthermore, the discrepant biological hypotheses and sites of PRP administration resulted in diverse and incomparable radiologic outcomes among studies, including graft remodeling, tunnel enlargement, and harvest site healing.^14,16^ In terms of graft remodeling, only 1 RCT^56^ with intra-articular injection and 1 RCT^57^ with intratunnel and intragraft injection reported improved short-term MRI results at 4 to 6 months and 4 to 6 weeks after ACLR, respectively, while the statistical significance diminished over time.

Preclinical studies have explored a wide range of biological augmentation (eg, growth factors, stem cells, and biomaterials) for tendon-to-bone healing after ACLR.^17^ With regard to PRP, the discrepancy between promising biological effects in animal studies^58,59^ and limited clinical efficacy in RCTs may be attributed to the persistence and complexity of graft remodeling in humans, where the MRI signal intensity peaks at 6 months while the complete maturation process lasts for more than 2 years (compared with several weeks to months for sheep).^5,6,41^

Given the consensus that single-dose intraoperative PRP injection was ineffective in long-term outcomes of ACLR, it has been necessary for subsequent studies to modify the conventional protocols, such as timing and dosage of injection. Based on the hypothesis of improving knee symptoms and function by continuously promoting graft healing and remodeling, we designed a 3-dose monthly interval protocol for postoperative intra-articular injection of liquid-form, delayed activated, and leukocyte-poor PRP.^19,60,61^ To our knowledge, this RCT is the first high-level study of the effects of postoperative PRP injection in patients undergoing ACLR, and standardized surgical procedures, rehabilitation programs, and intervention protocols were performed to lower the risk of confounding bias. The negative results of our study were also consistent with previous RCTs using 2 to 3 doses of postoperative PRP injection at weekly or monthly intervals for patients with rotator cuff tears or ankle instability.^21,62^ On the basis of current findings, postoperative PRP injection should not be advocated in patients undergoing ACLR unless new evidence emerges. Nevertheless, further studies may focus on identifying patients at a high risk of biological failure^55^ and determining rigorous indications for PRP or other biological augmentation.

### Limitations

This study has several limitations. First, participants were not masked to the group allocation, and the control group did not receive saline injection; the assessments of PROs may be affected by subjective tendency and the placebo effect. As a remedy, radiologic evaluation and physical examinations were performed with allocation concealment from investigators. Second, although our sample included participants treated at a national medical center using standardized protocols, generalizability of the results may be limited for clinicians applying different surgical techniques or treatment protocols. Third, there were difficulties in quantifying the compliance and completion of rehabilitation programs and assessing the intergroup imbalance. Fourth, the analyses of whole blood and PRP were limited to blood cell counting, while the concentrations of growth factors and cytokines were not analyzed for participants in this trial. Fifth, the follow-up duration was relatively short. Although a 12-month follow-up is considered long enough for studies on PRP,^13,14^ the progression of graft maturation and return to sports usually lasts for years,^5,6^ and a longer follow-up period may provide more information.

## Conclusions

In this RCT among patients undergoing ACLR, the addition of postoperative intra-articular PRP injection did not result in superior improvement in knee symptoms and function at 12 months compared with no postoperative injection. Further studies are required to determine appropriate indications for PRP in musculoskeletal disorders.

## Notes

Supplement 1.
Trial Protocol

Supplement 2. **eTable 1.** Processing, Characteristics, and Delivery of Platelet-Rich Plasma **eTable 2.** Primary and Sensitivity Analyses of KOOS_4_ During Follow-Up Visits **eTable 3.** Multivariable Linear Regression Analysis of KOOS_4_ at 12 Months **eTable 4.** Physical Examinations During Follow-Up Visits (Primary Analysis) **eTable 5.** Patient-Reported Outcomes During Follow-Up Visits (Per-Protocol Analysis) **eTable 6.** Graft Maturity on MRI During Follow-Up Visits (Per-Protocol Analysis) **eTable 7.** Physical Examinations During Follow-Up Visits (Per-Protocol Analysis) **eTable 8.** Medications and Adverse Events During Follow-Up Visits

Supplement 3.
Data Sharing Statement
