# Supplementary material for: Intra-Articular Platelet-Rich Plasma Injection After Anterior Cruciate Ligament Reconstruction: A Randomized Clinical Trial
Source: JAMA Netw Open. 2024 May 10;7(5):e2410134. doi: 10.1001/jamanetworkopen.2024.10134 (PMC11087838; doi:10.1001/jamanetworkopen.2024.10134)
Supplement: Supplement 1. — Trial Protocol [file jamanetwopen-e2410134-s001.pdf]

Trial Protocol and Statistical Analysis Plan

Contents

Section 1. Introduction — P2

Section 2. Purposes — P3

Section 3. Study Design — P3

Section 4. Study Population — P3

Section 5. Sample Size — P5

Section 6. Participant Recruitment — P5

Section 7. Group Allocation — P8

Section 8. Intervention and Control — P9

Section 9. Follow-up Visits — P10

Section 10. Outcome Measures — P11

Section 11. Adverse Events — P13

Section 12. Statistical Analysis — P14

Section 13. Ethical Procedures — P16

Section 14. Data Management — P17

Section 15. Quality Assurance — P17

Section 16. References — P17

Note: The original protocol with statistical analysis plan was submitted to the institutional review board of Shanghai Sixth People’s Hospital on February 18, 2021, and the formal approval was obtained on March 12, 2021.

Study Protocol

Title:

The Effect of Postoperative Injection of Platelet-Rich Plasma on Knee Edema and Adhesion, and Graft Revascularization

Institution(s) of Research: Shanghai Sixth People's Hospital

Principal Investigator: Jinzhong Zhao

Institution of Ethical Review: Shanghai Sixth People's Hospital

Date of Version: 2021-02-18

Contact Information: jzzhao@sjtu.edu.cn

Section 1. Introduction

Anterior cruciate ligament (ACL) injury is one of the most common sports injuries of the knee joint. There is an increasing incidence of ACL injuries, with approximately 60,000 to 200,000 per year in the United States, especially for teenagers.[1] With the incidences of sports injuries increasing gradually in China, the public have paid more attention to the functional recovery of sports activities, and ACL reconstruction (ACLR) has become a conventional surgery performed in sports medicine. In ACLR, autologous tendons are usually harvested and prepared into several-stranded grafts, which are passed through the tibial and femoral tunnels and fixed by suspension or extrusion.[2] Afterwards, the grafts undergo a series of biological tendon-to-bone healing processes (termed "ligamentization", a transformation from tendons to the functional ACL), including avascular necrosis in the early healing stage, proliferation and revascularization in the remodeling stage, and restoration of histological and biomechanical properties in the maturation stage.[3,4] Factors associated with unfavorable graft remodeling and maturation (biological recovery) after ACLR include graft impingement, sports reinjury, and residual knee laxity.[5] Furthermore, edema and adhesions are common postoperative reactions, causing symptoms of knee swelling, pain, and limited range of motion as well as hampering the functional recovery. To prevent the development of adverse outcomes (such as graft failure, arthrofibrosis, and osteoarthritis), timely intervention should be taken, including medications, physical therapy, and arthroscopic surgeries.[6]

Platelet-rich plasma (PRP) is a biological product made from autologous blood through centrifugation and degranulation steps, with good records of safety and promising efficacy in therapy for musculoskeletal disorders. The platelet concentration of PRP is about 3–5 times that of whole blood, with enrichment of growth factors and cytokines; hence, PRP is widely recognized to have a potential for promotion of healing after tissue injury or repair, which has attracted the interest of researchers in orthopedics and sports medicine.[7] Cell and animal studies have shown that PRP can promote skeletal muscle regeneration, reduce cell apoptosis, regulate tissue differentiation, and inhibit adhesion and fibrosis.[8-10] When it comes to clinical application, the actual effect of PRP is controversial. Systematic reviews have demonstrated that PRP injection resulted in clinical improvements in patients with lateral epicondylitis and knee osteoarthritis;[11,12] with regard to ACLR, the application of PRP-pretreated grafts was ineffective in promoting graft-to-bone healing and preventing tunnel widening,[13,14] while intraoperative injection of PRP in the graft harvesting area and bone tunnels may help reduce donor site pain and promote graft revascularization, respectively.[15,16] It can be seen that the actual effect of PRP depends on the timing, locations, and methods of administration. Based

on the promising biological effects and good safety records of PRP in basic scientific research and clinical practice, we hypothesize that injecting the PRP into the joint cavity at fixed intervals after ACLR may result in reduction in knee symptoms related to edema and adhesion reactions as well as promotion of graft revascularization.

In terms of research feasibility, the principal investigator is an experienced orthopedic surgeon specialized in knee arthroscopic surgeries and expertized in clinical study design; the annual amount of ACLR surgeries performed in the department of sports medicine in our institution is at the leading level throughout the country; the standard operating procedures (SOP) for ACLR, postoperative rehabilitation, and PRP preparation and injection have been formulated and generalized according to high-level evidence as well as clinical practice. Investigators in the research group have been engaged in basic and clinical studies related to ACL injury and reconstruction for many years, with proficiency in patient recruitment, informed consent, follow-up visiting, outcome measurements and statistical analyses.

Section 2. Purposes

The main purpose of this study is to evaluate the clinical efficacy of postoperative intra-articular PRP injection on subjective outcomes (overall perception of pain, symptoms, function in sports activities, and quality of life) in patients with ACLR.

The secondary purposes of this study are to evaluate the various clinical and radiological outcomes (including pain, swelling, adhesion, and graft revascularization and maturation) during the first year after ACLR with or without postoperative intra-articular PRP injection.

Section 3. Study Design

This study is a prospective, single-blinded, randomized controlled trial to perform in a single center (Department of Sports Medicine, Shanghai Sixth People’s Hospital).

Section 4. Study Population

4.1 Inclusion Criteria

Participants included in the study should meet all the following criteria:

① Patients with ACL injury diagnosed by radiology or under arthroscopy who are scheduled to undergo ACLR;
② Aged 16–45 years old with epiphyseal closure of the femur and tibia;
③ The range of motion of the affected knee is nearly normal before the operation (reaching nearly full knee extension and bending over 120°);
④ Volunteer to participate in the research and sign the informed consent.

4.2 Exclusion Criteria

Patients screened for eligibility should be excluded if they meet any of the following criteria:

① Multiple ligament injuries caused by severe trauma (including posterior cruciate ligament injury, posterolateral complex injury, medial patellofemoral ligament injury, and grade 2 or 3 medial collateral ligament injury);
② Bilateral ACL injuries or contralateral knee dysfunction;
③ Combined with tibial plateau fracture, severe cartilage damage or defect, meniscus repair, or knee osteoarthritis;
④ History of the knee joint surgery (ipsilateral and contralateral);
⑤ Combined with systemic diseases, such as malignant tumors, blood system diseases, rheumatic diseases, and endocrine or metabolic diseases;
⑥ History of infectious diseases transmitted through blood, such as an infection of human immunodeficiency virus, hepatitis B or C virus, or treponema pallidum;
⑦ Children under 16 years old, or women in pregnancy and lactation;
⑧ History of drug and alcohol abuse, cognitive dysfunction, or mental illness;
⑨ Other conditions that affect the cooperation with postoperative treatment, rehabilitation, and completion of follow-up visits.

4.3 Criteria of Protocol Violation

Participants included in the study should be classified as protocol violation (not excluded in the intention-to-treat analysis but excluded in the per-protocol analysis) if they meet any of the following criteria:

① Participants in the intervention group who fail to receive the intervention protocols (receiving 0, 1, 2, or >3 doses, or 3 doses with violation of injection schedules);
② Participants in both groups who undergo reoperation (including debridement, lysis of adhesion, revision ACLR, and other knee surgeries) or receive knee injection out of the protocol (including PRP, sodium hyaluronate, and other medications).

4.4 Criteria of Dropout

Participants included in the study should be classified as dropout (with missing data but not excluded in the analyses) if they meet any of the following criteria:

① Lost to follow-up during the study period;

② Request to withdraw the informed consent;

③ Other circumstances that affect the completion of follow-up visits.

Section 5. Sample Size

The minimal clinically important difference (MCID) of the primary outcome varies among studies, ranging from 8 to 40 for each subscale among patients with knee injuries or osteoarthritis.[17] Based on the anchor-based method, the MCIDs are 10.7–16.7 for the 4 subscales, averaged 13.8.[18]. Therefore, the statistical hypothesis is based on detecting a between-group difference of 13 points. The maximum SD is estimated to be 20 points,[19] thus the effect size is 0.65. With the 2-sided significance level and power setting at 0.05 and 0.9, respectively, a sample size of 51 participants is required in each group (1:1 allocation, calculated using G*Power 3.1, based on the test of mean difference between two independent groups, and assuming a balanced baseline). After adding the dropout (estimated within 15%), a total of 120 participants (60 in each group) should be included in this study.

Section 6. Participant Recruitment

6.1 Inclusion and Exclusion

Participants who are scheduled to undergo ACLR and hospitalized in the department of sports medicine in our institution will be screened for eligibility. After checking the inclusion and exclusion criteria, the study information will be introduced to eligible patients. For patients who are interested in participating the trial, oral informed consents will be carried out and written informed consents will be signed if they decide to participate. The oral and written informed consents will include detailed protocols of intervention and outcome measures, as well as potential benefits and risks throughout the trial. Patients are informed that the conventional surgical and rehabilitation treatment (following the SOP) as well as follow-up visits will not be affected no matter whether they decide to participate or not.

In particular, the inclusion and exclusion criteria involve demographic characteristics, injury patterns, medical history, and surgical details. Although most criteria can be checked before surgery, cartilage defect and meniscal treatment should be confirmed under arthroscopy during surgery. The exclusion criteria will be rechecked after surgery to exclude patients with severe cartilage defect or meniscal repair (because of distinguished rehabilitation programs and objectives from isolated ACLR or ACLR with meniscal resection).

6.2 Baseline Evaluation

① Demographic characteristics (self-reported, before surgery): age, gender, body mass index, affected side, Beighton score (hypermobility), preinjury Tegner score (sports level),[20] types of sports participation at injury, and time from injury to surgery.

② Subjective assessments (in written forms, before surgery): Knee Injury and Osteoarthritis Outcome Score (KOOS),[21] P_4 score for pain,[22] Lysholm score,[23] and subjective International Knee Documentation Committee (IKDC) score.[24]

③ Physical examinations: knee range of motion[25] and knee circumference at the mid-patellar level[26] (before surgery); knee laxity[27] (under anesthesia, during surgery).

④ Surgical details: amount of ACL remnant,[28] graft diameters (for anteromedial and posterolateral bundles), and meniscal treatment (none or meniscal resection).

6.3 Surgical Procedures

Participants included in this study will receive conventional anatomic double-bundle ACLR using hamstring autograft following the institutional SOP:

① Position and diagnostic arthroscopy

The patients is in the supine position, with the affected knee flexed at 90°, and a baffle is placed on the outside of the proximal thigh. The arthroscope is inserted from the anterolateral approach to observe the ACL injury and confirm the diagnosis.

② Autograft harvest and preparation

Through the skin and sartorius aponeurosis incisions, the autologous semitendinosus tendon (ST) and gracilis tendon (GT) are recognized, ligated, and torn off the tibial insertions with the periosteum. After cutting the connecting fibers, the ST and GT are harvested using a tendon stripper. The ST and GT are braided and folded into two 4-stranded grafts, which are separately passed through a non-slotted diameter measurement tool.

③ Tunnel positioning and creation

The femoral and tibial footprint centers of the ACL are located and marked using a radiofrequency probe. For each bundle of the ACL, a tunnel positioner is inserted from the anteromedial approaches and positioned at the tibial attachment, and the guide pin is drilled through the positioner towards the femoral attachment. The tibial and femoral tunnels of the anteromedial and posterolateral bundles are sequentially created using the guide pin.

④ Proximal and distal graft fixation

The proximal ends of the 4-stranded ST and 4-stranded GT are separately pulled from the tibial tunnels through the joint cavity to the femoral tunnels. The suture tapes on the proximal graft ends are fixed on cortical buttons over the outer orifices of the femoral tunnels. With the patient at full knee extension and the distal suture ends tensioned, the distal graft ends are fixed using an interference screw squeezed into the tibial tunnel as well as a cortical button with an adjustable loop setting through a transtibial ridge tunnel. Finally, the sartorius aponeurosis is repaired and the incisions and approaches are closed.

6.4 Rehabilitation Programs

Participants included in this study will receive postoperative rehabilitation education orally, in written forms, and through WeChat online videos following the institutional SOP:

① At 1–6 weeks (goals: swelling reduction; normal walking; knee flexion >90°)

Patients are allowed to bear weight with an ACL brace locking at full extension as tolerated immediately after the operation. Patients should abandon the walking stick and brace by 2 and 4 weeks, respectively. Muscle strength exercises include ankle pumps, isometric contraction of quadriceps, straight leg raises, and calf raises. Range of motion exercises include patellar mobilizations, heel slides, seated assisted flexion, and leg pressing.

② At 7–12 weeks (goals: flexion >120°; extensor strength and proprioception training)

Patients should continue carrying out flexion and extension exercises (active assisted → active). Muscle strength exercises include squatting (0–45°) and resistive knee extension (0–5 kg). Flexibility exercises include step-ups/downs and stationary bikes.

③ At 4–6 months (goals: flexor strength and balance training; running/non-pivoting sports)

Patients should aim at full knee flexion (if not reached yet) and extension (rather than hyperextension). Training programs include step-ups/downs, resistive knee flexion (0–10 kg), squatting (double-leg → single-leg), single-leg hops, and steady running.

④ At 7–12 months (goals: strength and agility training; noncontact pivoting sports)

Training programs include single-leg hops for distance, side slides, shuttle sprints, side crossing steps, and specific training for targeted sports. Patients are encouraged to return to pivoting sports by 9 months, depending on radiological findings and sports readiness.

Section 7. Group Allocation

7.1 Randomization

Prior to the beginning of participant enrollment, a random number sequence with 120 numbers (with 60 odd numbers for allocation to the intervention group and 60 even numbers for allocation to the control group) will be generated by computer. Then, the 120 sheets (each printed with one random number as generated) will be separately and sequentially sealed in 120 numbered opaque envelopes. The number sequence for group allocation will not be predictable. An independent trial assistant will be responsible for generating random numbers and keeping the envelopes. This assistant should be blocked contact with participants and investigators who should be concealed to the group allocation in this study.

For each participant, the randomization should be performed after surgery; otherwise, the postoperative exclusion may cause imbalanced allocation, and the risk of allocation leakage to surgeons may increase. Once the participant is rechecked for inclusion and exclusion criteria and given the enrollment and randomization numbers (corresponding to the envelopes), the group allocation (intention-to-treat) will not be changed.

7.2 Allocation Concealment

In a preliminary survey about motivation to participate, we introduced 2 patterns of allocation concealment to patients with ACLR: (1) double-blinded: all participants receive blood withdrawal, while only participants in the intervention group would receive real PRP injection; (2) single-blinded: participants in both groups are aware of group allocation, and only participants in the intervention group would receive the blood withdrawal and PRP injection. The results showed that 9 in 10 vs 2 in 10 respondents were willing to participate in the single- and double-blinded trials, respectively. Compared to traditional double-blinded designs with placebo control, the control group in PRP studies would receive additional blood withdrawal without any potential therapeutic benefits. Given the motivation for participation and potential ethical concerns, participants will be aware of their own group allocation.

The investigators who have the accessibility to treatment, outcome measurements, and data curation should be blinded throughout the study, including the surgeons, physical therapists, clinicians for informed consent, data entry, physical examinations, and radiological measurements, as well as the statistician. Participants will be instructed not to disclose their group allocation to these investigators before each follow-up visit. For each participant, the participant contacting assistant will be responsible for coordinating follow-up visits, taking the opaque envelope from the independent randomization assistant, and revealing the group allocation to the participant at the first follow-up visit. This assistant should be inaccessible to the treatment, outcome measurements, and data curation processes.

Section 8. Intervention and Control

8.1 Intervention

Participants allocated to the intervention group will receive blood withdrawal followed by PRP injection at 4 weeks (± 3 days), 8 weeks (± 5 days), and 3 months (± 7 days) during the routine follow-up visits (after clinical assessments) following the standardized protocol:

① Materials for treatment

PRP preparation kit (containing a 50-mL syringe, three 20-mL syringes, 2 centrifuge tubes, 2 pipettes, 5 mL of Anticoagulant Citrate Dextrose Solution A, and several puncture needles), centrifuge (maximum speed, 4000 r/min; maximum force, 2826 g), automated hematology analyzer, and conventional injection equipment.

② Whole blood withdrawal

After skin sterilization, 45 mL of venous blood is drawn from the antecubital fossa into a 50-mL syringe with 5 mL of anticoagulant preloaded in the syringe.

③ PRP preparation

The 50 mL of whole blood is transferred into a centrifuge tube and centrifuged at 260 g for 10 minutes. After centrifugation, the sample is separated into 3 layers (from bottom to top: red blood cells, buffy coat containing, and acellular plasma). The upper and middle layers are aspirated into a new centrifuge tube using a pipette. The sample is recentrifuged at 360 g for 15 minutes and separated into 2 layers. Then, a new pipette is used to aspirate the supernatant (platelet-poor plasma) and leave 5 mL of leukocyte-poor PRP in the tube. Blood cell counts of whole blood and PRP are analyzed using an automated hematology analyzer.

④ Intra-articular injection

The PRP should be injected into the knee within 45 minutes after blood withdrawal. With the patient in the sitting position and the knee flexed at 90°, the injection needle is punctured through the knee anterolateral approach (lateral to the patellar tendon and superior to the joint line) towards the intercondylar notch under ultrasound guidance. After injection, passive knee flexion and extension are implemented for 10 cycles. Patients are instructed to rest for 30 minutes in the treatment room for observation of adverse events.

8.2 Control

Participants allocated to the control group will undergo the same timing and assessments of follow-up visits without postoperative knee injection. Participants in both groups should avoid co-interventions which may cause confounding biases, including unscheduled knee injection and non-steroidal anti-inflammatory drugs, while acetaminophen can be prescribed for participants who report a moderate to severe pain if required (should be suspended for 7 days before clinical assessments). If participants receive additional medications or treatment out of the protocol, they should report them completely to the clinicians at follow-up visits.

Section 9. Follow-up Visits

	Pre-op	Post-op	4 wk	8 wk	3 mo	6 mo	12 mo
Participant recruitment							
Eligibility screening	√						
Informed consent	√						
Data collection	√						
Rehabilitation education		√					
Randomization		√					
PRP injection by group							
Intervention group	×	×	√	√	√	×	×
Control group	×	×	×	×	×	×	×
Outcome measures							
Primary outcome	√						√
Subjective assessments	√				√	√	√
Radiological scanning	√				√	√	√
Physical examinations	√		√	√	√	√	√
Adverse events	√		√	√	√	√	√

Section 10. Outcome Measures

10.1 Primary Outcome

The primary outcome of this study is the $KOOS_4$ score at 12 months postoperatively. The KOOS has been developed for evaluating knee symptoms and function in a variety of knee injuries and osteoarthritis and validated in ACL-injured and ACL-reconstructed patients.[21,29] KOOS includes 42 items covering 5 subscales: pain (9 items), symptoms (7 items), function in activities of daily living (17 items), function in sports and recreation (5 items), and quality of life (4 items). For each item, the responses are presented in 5-point Likert scales (range, 0–4). For each subscale, the overall scores are summed by each item and standardized to range from 0 to 100, with higher scores indicating fewer symptoms or better knee function (100 indicating best knee function without symptoms). Given that patients with ACL injuries and ACLR have relatively high scores of the function in activities of daily living subscale, some high-level studies have introduced $KOOS_4$, which is calculated as the average score of the other 4 subscales (pain, symptoms, function in sports and recreation, and quality of life), to avoid the ceiling effect.[30,31] Based on the anchor-based MCIDs for each subscale of the KOOS,[18] the MCID for $KOOS_4$ is determined as 13 points in this study.

10.2 Secondary Outcomes: Subjective Assessments

① Global Rating of Change (GROC) scale,[32] at 12 months

GROC scale assesses the overall change of condition resulting from the treatment (including the surgery and postoperative treatment). Scores range from −7 (a very great deal worse) to +7 (a very great deal better), with higher positive values indicating more improvement, and scores ≥+3 (somewhat better) are considered as clinically meaningful.

② KOOS subscales, at 3, 6, and 12 months

The MCIDs for KOOS subscales of pain, symptoms, activities of daily living, sports and recreation, and quality of life are 16.7, 10.7, 18.4, 12.5, and 15.6, respectively.[18]

③ P_4 score for pain,[22] at 3, 6, and 12 months

The visual analog scale (VAS) for pain is assessed on a straight horizontal line; scores range from 0 (no pain) to 10 (worst pain possible), with 1−3 indicating mild pain, 4−6 indicating moderate pain, and 7−10 indicating severe pain. To comprehensively evaluate the pain in different timing and conditions, the P_4 score is calculated as the sum of VAS scores at 4 timepoints (morning, afternoon, evening, and with activity) during the past 2 days.

④ Tegner score,[20] at 3, 6, and 12 months

Tegner Activity Scale assesses the highest level of current sports participation. Scores range from 0 (sick leave or disability) to 10 (professional level of competitive sports), with higher values indicating higher levels of sports activities, and scores ≥6 are considered as return to pivoting sports. The MCID (as minimally detectable) for Tegner score is 1.[18]

⑤ Lysholm score,[23] at 3, 6, and 12 months

Lysholm Knee Questionnaire is specifically designed for ligament injuries and includes 8 items regarding subjective perceptions (such as instability, pain, and locking). Scores range from 0 to 100, with higher values indicating fewer symptoms and better knee function in daily living. The MCID for Lysholm score is 10.7 (distribution-based, from our data).

⑥ Subjective IKDC score,[24] at 3, 6, and 12 months

IKDC Subjective Knee Form includes 18 items covering 3 domains (symptoms, sports activities, and function). Scores range from 0 to 100, with higher values indicating better knee conditions. The MCID for IKDC score is 10.4 (distribution-based, from our data).

10.3 Secondary Outcomes: Radiological Evaluation

Participants included in this study will undergo magnetic resonance imaging (MRI) scanning at 3, 6, and 12 months. A 3.0-T MRI unit (MAGNETOM Prisma, Siemens) is selected for standardization of the radiological evaluation. The institutional protocol of conventional sequences for knee MRI include axial, sagittal, and coronal planes of T1-weighted imaging and fat-saturated proton-density-weighted imaging (FS-PDWI), with a slice thickness of 3 mm. In particular, the sagittal plane is determined by the vertical plane of the line tangent to the posterior cortices of medial and lateral femoral condyles on the axial plane.

The graft maturity will be evaluated on sagittal FS-PDWI sequences (repetition time, 3010 ms; echo time, 44 ms) using 0.1-cm^2 circular regions of interest (ROIs) for measuring the signal intensity. For the signals of background and quadriceps tendon, the ROIs are placed at 2 cm anterior to the patellar tendon and patellar upper limit level, respectively.[33] For the signals of ACL graft, the anteromedial and posterolateral bundles are both separated into 3 sections: (1) femoral intra-tunnel graft, (2) intra-articular graft, and (3) tibial intra-tunnel graft.[34] In each section, 3 ROIs are selected to calculate the mean signal. Signal-to-noise quotients (SNQ) are separately calculated for the 6 sections of the ACL graft using the following formula:

$$\mathrm{SNQ} = \frac{\text{Signal of ACL Graft} - \text{Signal of Quadriceps Tendon}}{\text{Signal of Background}}$$

All measurements will be performed on RadiAnt DICOM Viewer by 2 independent investigators who are blinded to group allocation. The interrater reliability will be evaluated using the intraclass correlation coefficient (ICC), with ICCs ≥0.75 indicating good reliability. The signal intensity on FS-PDWI usually reflects water content and vascularity, with lower SNQ values indicating a latter period of graft remodeling and better graft maturity.[35]

10.4 Secondary Outcomes: Physical Examinations

① Knee range of motion,[25] at each follow-up

The active-assisted range of motion is measured by placing the axis of a goniometer over the lateral femoral epicondyle and lining the two arms along the femoral and fibular axes, with positive values indicating knee flexion and negative values indicating hyperextension. The knee flexion and extension are assisted by both arms of the participant (in the sitting position) and a firm pillow beneath the heel (in the supine position), respectively.

② Knee circumference,[26] at each follow-up

The knee circumference is measured by placing a tape measure circumferentially around the knee at the mid-patellar level while the participant is in the supine position with full knee extension. Results are presented as the side-to-side differences by subtracting the circumference of the contralateral knee, with higher positive values indicating worse swelling.

③ Knee laxity,[27] at 12 months

The anterior drawer and Lachman tests are used to assess knee anteroposterior laxity at 90° and 30° of flexion by manual, respectively; grades include 0 (0−2 mm), 1 (3−5 mm), 2 (6−10 mm), and 3 (>10 mm). The pivot-shift test is used to assess knee rotatory laxity by manual; grades include 0 (negative), 1 (glide), 2 (clunk), and 3 (gross reduction). A KT-1000 arthrometer is used for quantitative measurements of anterior tibial translation at 30° of knee flexion, with higher results indicating increased knee anteroposterior laxity.

Section 11. Adverse Events

11.1 Definition and Classification

Adverse event (AE) refers to any unfavorable and unintended sign (including an abnormal laboratory finding), symptom, or disease temporally associated with the use of a medical treatment or procedure in a patient or clinical investigation subject, whether or not related to the medical treatment or procedure. AEs are usually graded by the severity:

① Mild AEs: asymptomatic or mild symptoms; observations only; intervention not indicated.
② Moderate AEs: minimal or local intervention indicated; limiting instrumental activities.
③ Serious AEs (SAEs): AEs that (1) results in death, (2) is life-threatening, (3) requires inpatient hospitalization or causes prolongation of existing hospitalization, (4) results in persistent or significant disability, (5) may have caused a congenital anomaly or birth defect, or (6) requires intervention to prevent permanent impairment or damage.

In this study, the AEs are classified into intervention-related AEs (only occurrence in intervention group and probably related to the intervention) and common AEs (occurrence in both groups, whether or not increased with intervention). Possible intervention-related AEs include fainting, allergic reaction, pain at injection site, knee swelling, decreased range of motion, and infection at injection site. Because of the study population (patients with ACL injuries and undergoing ACLR), common AEs should include all complications and unfavorable outcomes accompanied with ACL injuries as well as those with ACLR, such as graft failure, residual laxity, decreased range of motion, and arthrofibrosis.

11.2 Record and Report

Once an AE occurs, the time of occurrence, clinical manifestation, treatment process and duration, relation with intervention, and outcome of the AE should be recorded in detail on the case report form. After an unintended sign (such as laboratory test abnormalities) occurs, the patient must be followed up until the test results return to normal, or return to pre-intervention levels, or the AE is determined to be unrelated to the intervention. In cases of SAEs, an SAE form must be filled in and reported to the institutional review board (IRB) (within 24 hours) and National Medical Products Administration (if applicable).

Section 12. Statistical Analysis

12.1 Primary Analysis

The primary analysis will be performed following the intention-to-treat principle, which emphasizes on the original group allocation by randomization. Specifically, once a participant is enrolled and undergoes randomization, the baseline and outcome measures will be included in the allocated group, regardless of whether the participant violates the protocols and whether the participant is dropout during follow-up: (1) if a participant is randomized to the intervention group but does not receive 3 doses of PRP injection, this participant will not be

excluded from the intervention group; (2) if a participant is randomized to the control group but receives knee injection out of the protocol, this participant will not be excluded from the control group; (3) if a participant is randomized to either group but lost to follow-up, this participant will not be excluded from the primary analysis, either. In principle, the primary analysis should include all participants randomized in this study. For the missing data, simple or multiple imputation will not be performed in the primary analysis.

All statistical analyses will be performed using the IBM SPSS software, with a 2-sided significance level of 0.05. Descriptive statistics will be presented as mean and SD (for normally distributed values), median and interquartile range (for non-normally distributed values), or number and percentage (for categorical variables).

① Subjective assessments

The primary outcome, $KOOS_4$ at 12 months, has been validated to be associated with knee symptoms and pain at baseline.[30] Referenced to statistical methods in previous studies,[31,36] an analysis of covariance (ANCOVA) will be performed to analyze the between-group difference using the baseline $KOOS_4$ as the covariate for adjustment. For other subjective assessments included in the secondary outcomes, ANCOVA models will also be applied to analyze the between-group differences with adjustment for the corresponding baseline scores. Between group differences will be presented as mean, 95% confidence interval (CI), and P value. For an outcome with a statistically significant difference, the clinical significance should be evaluated by comparing the mean and 95% CI with the MCID.

② Radiological evaluation

Although some studies have demonstrated that SNQ values may be normally-distributed and used Student t test for comparison, it should be noted that SNQ values are relative values (rather than absolute values such as KOOS and Tegner score) with high variability,[33,34] while analyses of between-group differences by subtracting 2 quotients is not clinically meaningful (at least not determined for now). Therefore, the graft maturity (represented by the SNQ values averaged for the 2 radiological investigators) will be considered as ordinal variables and compared using the non-parametric test (Mann-Whitney-Wilcoxon test).

③ Other variables

The Kolmogorov-Smirnov test will be applied to evaluate the normality for continuous variables. The Student t test will be used for comparison of continuous variables with normal distribution. The Mann-Whitney-Wilcoxon test will be used for comparison of non-normally distributed continuous variables and ordinal variables (including GROC, amount of ACL

remnant, and grades of knee laxity). The Pearson chi-square test or Fisher exact test will be used for comparison of categorical variables (including demographic data and adverse events), depending on expected frequencies for the contingency tables.

12.2 Post-hoc Sensitivity Analyses

For all outcomes with missing data, the number, proportion, and reasons of patients who are not available for the outcome should be reported. For any outcome with missing data >5%, multiple imputation by chained equations (fully conditional specifications method) should be performed with 20 iterations to evaluate the bias caused by non-random missing and detect any change towards a different direction of the significance level. Variables in the imputation model should include group allocation, age, gender, sports participation, graft diameters, meniscal treatment, and baseline $KOOS_4$ (or corresponding baseline evaluation, if applicable). These variables are potential predictors and confounders of clinical outcomes and may also be explored in multivariate linear regression analyses to quantitatively compare the effects of different factors associated with the clinical outcomes.

Furthermore, per-protocol (as-treated) analyses for all outcomes should be performed based on the criteria of protocol violation. In this context, participants who receive reoperation or any unscheduled knee injection (more, less, or violation of injection schedules) should be excluded from the originally randomized group. In particular, if a participant is found out to receive the intervention or control following exactly the same protocol as the opposite group, this participant should be included in the opposite group. Per-protocol analyses will follow the above-mentioned statistical methods in the primary analysis.

Section 13. Ethical Procedures

This study has been prospectively registered on Chinese Clinical Trial Registry (Identifier: ChiCTR2000040262) on November 26, 2020. This protocol, written informed consent, and other related documents is submitted to the IRB, and the first participant will be recruited only after the study protocol is approved. The investigators must submit an annual report on their research to the IRB at least once a year after they start participant recruitment. When the study is suspended or completed, the investigators must notify the IRB in written reports about main results and a complete analysis of adverse events. The investigators must promptly report all changes in their research work to the IRB, and any amendment to the study protocol or written informed consent must be submitted to the IRB for review and approval.

The investigators must provide the participants or their legal representatives with an easy-to-understand informed consent form approved by the IRB and give them sufficient time to consider the participation. Participants should not be enrolled until the written informed consent is signed. All updated versions of the informed consent and other information should be provided to the participant during the study period. The informed consent should be retained for future review as an important document of the clinical trial.

Section 14. Data Management

The investigators should keep the data (including case report forms and related documents) properly in a fixed and locked site for future review. The results of this study may be published in a medical journal and/or presented at an academic conference, but the personal information of participants should be confidential in accordance with legal and ethical requirements. When necessary, staffs authorized by health administration and the IRB may have access to patient information in accordance with regulations.

Section 15. Quality Assurance

The principal investigator certified by Good Clinical Practice is responsible for study design and internal supervision. A total of 8–12 investigators will engage in major study processes, including informed consent, surgical operation, rehabilitation education, randomization, follow-up contact, PRP therapy, physical examination, radiological measurement, and statistical analysis. Critical quality assurance procedures include:

① Participants should undergo surgical procedures, rehabilitation programs, and PRP injection by certain therapists following standardized institutional protocols.

② Randomization and concealment of group allocation to surgeons, outcome assessors, and data analysts should be carried out following well-designed programs.

③ Subjective assessments, radiological measurements, and physical examinations should be completed by certain assessors following predetermined reliable methods.

Section 16. References

[1] Herzog M M, Marshall S W, Lund J L, et al. Trends in Incidence of ACL Reconstruction and Concomitant Procedures Among Commercially Insured Individuals in the United States, 2002-2014[J]. Sports Health, 2018, 10(6): 523-531.
[2] Zhao J, He Y, Wang J. Double-bundle anterior cruciate ligament reconstruction: four versus eight strands of hamstring tendon graft[J]. Arthroscopy, 2007, 23(7): 766-770.
[3] Claes S, Verdonk P, Forsyth R, et al. The "ligamentization" process in anterior cruciate ligament reconstruction: what happens to the human graft? A systematic review of the literature[J]. Am J Sports Med, 2011, 39(11): 2476-2483.
[4] Ménétrey J, Duthon V B, Laumonier T, et al. "Biological failure" of the anterior cruciate ligament graft[J]. Knee Surg Sports Traumatol Arthrosc, 2008, 16(3): 224-231.
[5] Somanathan A, Tandon A, Yang L W. Review of magnetic resonance imaging features of complications after anterior cruciate ligament reconstruction[J]. Singapore Med J, 2019, 60(2): 63-68.
[6] Sanders T L, Kremers H M, Bryan A J, et al. Procedural intervention for arthrofibrosis after ACL reconstruction: trends over two decades[J]. Knee Surg Sports Traumatol Arthrosc, 2017, 25(2): 532-537.
[7] Kia C, Baldino J, Bell R, et al. Platelet-Rich Plasma: Review of Current Literature on its Use for Tendon and Ligament Pathology[J]. Curr Rev Musculoskelet Med, 2018, 11(4): 566-572.
[8] Li H, Hicks J J, Wang L, et al. Customized platelet-rich plasma with transforming growth factor β1 neutralization antibody to reduce fibrosis in skeletal muscle[J]. Biomaterials, 2016, 87: 147-156.
[9] Xie X, Zhao S, Wu H, et al. Platelet-rich plasma enhances autograft revascularization and reinnervation in a dog model of anterior cruciate ligament reconstruction[J]. J Surg Res, 2013, 183(1): 214-222.
[10] Tsai W C, Yu T Y, Chang G J, et al. Platelet-Rich Plasma Releasate Promotes Regeneration and Decreases Inflammation and Apoptosis of Injured Skeletal Muscle[J]. Am J Sports Med, 2018, 46(8): 1980-1986.
[11] Le A D K, Enweze L, Debaun M R, et al. Current Clinical Recommendations for Use of Platelet-Rich Plasma[J]. Curr Rev Musculoskelet Med, 2018, 11(4): 624-634.
[12] Shen L, Yuan T, Chen S, et al. The temporal effect of platelet-rich plasma on pain and physical function in the treatment of knee osteoarthritis: systematic review and meta-analysis of randomized controlled trials[J]. J Orthop Surg Res, 2017, 12(1): 16.
[13] Davey M S, Hurley E T, Withers D, et al. Anterior Cruciate Ligament Reconstruction with Platelet-Rich Plasma: A Systematic Review of Randomized Control Trials[J]. Arthroscopy, 2020, 36(4): 1204-1210.

[14] Andriolo L, Di Matteo B, Kon E, et al. PRP Augmentation for ACL Reconstruction[J]. Biomed Res Int, 2015, 2015: 371746.
[15] Seijas R, Cuscó X, Sallent A, et al. Pain in donor site after BTB-ACL reconstruction with PRGF: a randomized trial[J]. Arch Orthop Trauma Surg, 2016, 136(6): 829-835.
[16] Vogrin M, Rupreht M, Dinevski D, et al. Effects of a platelet gel on early graft revascularization after anterior cruciate ligament reconstruction: a prospective, randomized, double-blind, clinical trial[J]. Eur Surg Res, 2010, 45(2): 77-85.
[17] Maltenfort M, Díaz-Ledezma C. Statistics In Brief: Minimum Clinically Important Difference-Availability of Reliable Estimates[J]. Clin Orthop Relat Res, 2017, 475(4): 933-946.
[18] Harris J D, Brand J C, Cote M P, et al. Research Pearls: The Significance of Statistics and Perils of Pooling. Part 1: Clinical Versus Statistical Significance[J]. Arthroscopy, 2017, 33(6): 1102-1112.
[19] Aga C, Risberg M A, Fagerland M W, et al. No Difference in the KOOS Quality of Life Subscore Between Anatomic Double-Bundle and Anatomic Single-Bundle Anterior Cruciate Ligament Reconstruction of the Knee: A Prospective Randomized Controlled Trial With 2 Years' Follow-up[J]. Am J Sports Med, 2018, 46(10): 2341-2354.
[20] Tegner Y, Lysholm J. Rating systems in the evaluation of knee ligament injuries[J]. Clin Orthop Relat Res, 1985, (198): 43-49.
[21] Roos E M, Roos H P, Lohmander L S, et al. Knee Injury and Osteoarthritis Outcome Score (KOOS)--development of a self-administered outcome measure[J]. J Orthop Sports Phys Ther, 1998, 28(2): 88-96.
[22] Spadoni G F, Stratford P W, Solomon P E, et al. The evaluation of change in pain intensity: a comparison of the P4 and single-item numeric pain rating scales[J]. J Orthop Sports Phys Ther, 2004, 34(4): 187-193.
[23] Lysholm J, Gillquist J. Evaluation of knee ligament surgery results with special emphasis on use of a scoring scale[J]. Am J Sports Med, 1982, 10(3): 150-154.
[24] Irrgang J J, Anderson A F, Boland A L, et al. Development and validation of the international knee documentation committee subjective knee form[J]. Am J Sports Med, 2001, 29(5): 600-613.
[25] Rheault W, Miller M, Nothnagel P, et al. Intertester reliability and concurrent validity of fluid-based and universal goniometers for active knee flexion[J]. Phys Ther, 1988, 68(11): 1676-1678.
[26] Kirwan J R, Byron M A, Winfield J, et al. Circumferential measurements in the assessment of synovitis of the knee[J]. Rheumatol Rehabil, 1979, 18(2): 78-84.

[27] Irrgang J J, Ho H, Harner C D, et al. Use of the International Knee Documentation Committee guidelines to assess outcome following anterior cruciate ligament reconstruction[J]. Knee Surg Sports Traumatol Arthrosc, 1998, 6(2): 107-114.

[28] Kim S J, Choi C H, Chun Y M, et al. Anterior Cruciate Ligament Reconstruction Using Bone-Patellar Tendon-Bone Autograft with Remnant Preservation: Comparison of Outcomes According to the Amount of Remnant Tissue[J]. J Knee Surg, 2019, 32(9): 847-859.

[29] Muller B, Yabroudi M A, Lynch A, et al. Defining Thresholds for the Patient Acceptable Symptom State for the IKDC Subjective Knee Form and KOOS for Patients Who Underwent ACL Reconstruction[J]. Am J Sports Med, 2016, 44(11): 2820-2826.

[30] Hamrin Senorski E, Svantesson E, Spindler K P, et al. Ten-Year Risk Factors for Inferior Knee Injury and Osteoarthritis Outcome Score After Anterior Cruciate Ligament Reconstruction: A Study of 874 Patients From the Swedish National Knee Ligament Register[J]. Am J Sports Med, 2018, 46(12): 2851-2858.

[31] Frobell R B, Roos E M, Roos H P, et al. A randomized trial of treatment for acute anterior cruciate ligament tears[J]. N Engl J Med, 2010, 363(4): 331-342.

[32] Guyatt G H, Norman G R, Juniper E F, et al. A critical look at transition ratings[J]. J Clin Epidemiol, 2002, 55(9): 900-908.

[33] Ahn J H, Lee S H, Choi S H, et al. Magnetic resonance imaging evaluation of anterior cruciate ligament reconstruction using quadrupled hamstring tendon autografts: comparison of remnant bundle preservation and standard technique[J]. Am J Sports Med, 2010, 38(9): 1768-1777.

[34] Zhang Y, Liu S, Chen Q, et al. Maturity Progression of the Entire Anterior Cruciate Ligament Graft of Insertion-Preserved Hamstring Tendons by 5 Years: A Prospective Randomized Controlled Study Based on Magnetic Resonance Imaging Evaluation[J]. Am J Sports Med, 2020, 48(12): 2970-2977.

[35] Weiler A, Peters G, Mäurer J, et al. Biomechanical properties and vascularity of an anterior cruciate ligament graft can be predicted by contrast-enhanced magnetic resonance imaging. A two-year study in sheep[J]. Am J Sports Med, 2001, 29(6): 751-761.

[36] Getgood A M J, Bryant D M, Litchfield R, et al. Lateral Extra-articular Tenodesis Reduces Failure of Hamstring Tendon Autograft Anterior Cruciate Ligament Reconstruction: 2-Year Outcomes From the STABILITY Study Randomized Clinical Trial[J]. Am J Sports Med, 2020, 48(2): 285-297.
