# Supplementary material for: Intra-Articular Platelet-Rich Plasma Injection After Anterior Cruciate Ligament Reconstruction: A Randomized Clinical Trial
Source: JAMA Netw Open. 2024 May 10;7(5):e2410134. doi: 10.1001/jamanetworkopen.2024.10134 (PMC11087838; doi:10.1001/jamanetworkopen.2024.10134)
Supplement: Supplement 2. — eTable 1. Processing, Characteristics, and Delivery of Platelet-Rich Plasma eTable 2. Primary and Sensitivity Analyses of KOOS4 During Follow-Up Visits eTable 3. Multivariable Linear Regression Analysis of KOOS4 at 12 Months eTable 4. Physical Examinations During Follow-Up Visits (Primary Analysis) eTable 5. Patient-Reported Outcomes During Follow-Up Visits (Per-Protocol Analysis) eTable 6. Graft Maturity on MRI During Follow-Up Visits (Per-Protocol Analysis) eTable 7. Physical Examinations During Follow-Up Visits (Per-Protocol Analysis) eTable 8. Medications and Adverse Events During Follow-Up Visits [file jamanetwopen-e2410134-s002.pdf]

Supplemental Online Content

Ye Z, Chen H, Qiao Y, et al. Intra-articular platelet-rich plasma after anterior cruciate ligament reconstruction: a randomized clinical trial. *JAMA Netw Open*. 2024;7(5):e2410134. doi:10.1001/jamanetworkopen.2024.10134

eTable 1. Processing, Characteristics, and Delivery of Platelet-Rich Plasma

eTable 2. Primary and Sensitivity Analyses of $KOOS_4$ During Follow-Up Visits

eTable 3. Multivariable Linear Regression Analysis of $KOOS_4$ at 12 Months

eTable 4. Physical Examinations During Follow-Up Visits (Primary Analysis)

eTable 5. Patient-Reported Outcomes During Follow-Up Visits (Per-Protocol Analysis)

eTable 6. Graft Maturity on MRI During Follow-Up Visits (Per-Protocol Analysis)

eTable 7. Physical Examinations During Follow-Up Visits (Per-Protocol Analysis)

eTable 8. Medications and Adverse Events During Follow-Up Visits

This supplemental material has been provided by the authors to give readers additional information about their work.

© 2024 Ye Z et al. *JAMA Network Open*.

eTable 1. Processing, Characteristics, and Delivery of Platelet-Rich Plasma[a]

Category	Checklist item	Detail
Processing	1. Whole blood withdrawal	Venous blood from antecubital fossa (45 mL)
	2. Anticoagulant	Acid Citrate Dextrose Solution A (5 mL), preloaded in the 50 mL syringe
	3. Whole blood storage	Processed immediately, no storage
	4. Commercial kit	WEGO PRP Preparation Kit[b]
	5. Centrifugation protocol	Stage 1: 260 g, 10 min (for whole blood)<br>Stage 2: 360 g, 15 min (for upper and middle layers separated in stage 1)
	6. PRP volume	5 mL (bottom layer separated in stage 2)
	7. PRP storage/processing	Delivered immediately, no storage
Characteristics	1. Whole blood cell analysis, mean±SD	Using an automated hematology analyzer<br>Erythrocyte, $(4.36\pm0.24)\times10^{12}$/L; leukocyte, $(6.20\pm0.99)\times10^{9}$/L; platelet, $(152\pm31)\times10^{9}$/L
	2. PRP cell analysis, mean±SD	Using an automated hematology analyzer<br>Erythrocyte, $(0.03\pm0.00)\times10^{12}$/L; leukocyte, $(4.60\pm0.70)\times10^{9}$/L; platelet, $(678\pm173)\times10^{9}$/L
	3. Platelet enrichment	4.5±0.8 times (range, 2.8 to 6.0)
	4. Leukocyte concentration	Reduced by 25% on average
Delivery	1. PRP format	Liquid (5 mL, no activating agent)
	2. PRP activation	Endogenous activation
	3. Administration route	Knee injection through an anterolateral approach and towards the intercondylar notch (sitting position, ultrasound guidance)
	4. Dosage	3 doses (at 4 wk, 8 wk, and 3 mo after ACLR)

Abbreviations: ACLR, anterior cruciate ligament reconstruction; PRP, platelet-rich plasma.

a. The reported items are designed according to the checklist of Minimum Reporting Requirements for Clinical Studies Evaluating Platelet-Rich Plasma. (Reference: Murray IR, Geeslin AG, Goudie EB, Petrigliano FA, LaPrade RF. Minimum Information for Studies Evaluating Biologics in Orthopaedics (MIBO): Platelet-Rich Plasma and Mesenchymal Stem Cells. *J Bone Joint Surg Am*. 2017;99(10):809-819.)

b. The WEGO PRP Preparation Kit (WEGO Ltd., Shandong, China) contains a syringe (50 mL) for blood withdrawal, 3 syringes (20 mL) for PRP preparation and injection, 2 centrifuge tubes, 2 pipettes, the anticoagulant (5 mL), and several puncture needles.

© 2024 Ye Z et al. *JAMA Network Open*.

eTable 2. Primary and Sensitivity Analyses of $KOOS_4$ During Follow-Up Visits[a]

Outcome, mean (SD)	PRP group	Control group	Adjusted between-group difference[b] (95% CI)	*P* value
Primary analysis[c]				
$KOOS_4$ at 3 mo	57.0 (16.1)	58.3 (17.9)	−0.4 (−6.3 to 5.6)	0.90
$KOOS_4$ at 6 mo	71.9 (14.1)	67.3 (14.5)	5.5 (0.4 to 10.6)	**0.03**
**$KOOS_4$ at 12 mo**	78.3 (12.0)	76.8 (11.9)	2.0 (−2.3 to 6.3)	0.36
Multiple imputation analysis[d]				
$KOOS_4$ at 3 mo	56.9 (15.9)	58.1 (17.8)	−0.5 (−6.4 to 5.3)	0.86
$KOOS_4$ at 6 mo	72.0 (14.0)	67.4 (14.5)	5.1 (0.1 to 10.2)	**0.045**
$KOOS_4$ at 12 mo	78.6 (11.9)	76.8 (11.9)	2.2 (−2.0 to 6.5)	0.30
Per-protocol analysis[e]				
$KOOS_4$ at 3 mo	57.3 (15.8)	58.9 (17.8)	−1.0 (−7.1 to 5.0)	0.73
$KOOS_4$ at 6 mo	73.0 (13.3)	68.1 (14.1)	5.3 (0.3 to 10.3)	**0.04**
$KOOS_4$ at 12 mo	79.4 (11.5)	76.9 (11.8)	2.7 (−1.6 to 7.0)	0.22

Abbreviations: KOOS, Knee Injury and Osteoarthritis Outcome Score; PRP, platelet-rich plasma.

a. The $KOOS_4$ is calculated as the average score for 4 subscales (pain, symptoms, sports and recreation, and quality of life) of the KOOS; scores range from 0 to 100, with higher scores indicating better knee-related results (patient-acceptable symptom state threshold, 70.9).

b. The between-group differences for $KOOS_4$ at follow-up visits are adjusted for the baseline $KOOS_4$ using an analysis of covariance model, with positive values indicating higher scores in the PRP group and negative values indicating higher scores in the control group.

c. The primary (intention-to-treat) analysis includes all randomized participants (60 in the PRP group and 60 in the control group). Six participants (3 in the PRP group and 3 in the control group) were lost to follow-up within 12 months, and the remaining 114 participants were available for the primary outcome ($KOOS_4$ at 12 months). $KOOS_4$ scores at 3 and 6 months were not prespecified in the trial protocol, and the results should be exploratory.

d. The multiple imputation (with 20 iterations) analysis is performed using the fully conditional specifications method, including group allocation, age, gender, sports participation, graft diameters, meniscal treatment, and baseline $KOOS_4$ in the model.

e. The per-protocol analysis includes 55 participants in the PRP group and 57 participants in the control group. The PRP group excludes 5 participants: 2 received 1 dose of PRP injection (unwilling to continue because of knee swelling and pain, as reported in adverse events), 1 received 2 doses of PRP injection (unwilling to continue because of time consumption), and 2 received reoperation (1 for arthrolysis because of limited range of motion and 1 for meniscal repair because of reinjury). The control group excludes 3 participants receiving reoperation (for arthrolysis because of limited range of motion).

© 2024 Ye Z et al. *JAMA Network Open*.

eTable 3. Multivariable Linear Regression Analysis of $KOOS_4$ at 12 Months[a]

Variable[b]	B (95% CI)[c]	Beta	*P* value
PRP injection	1.32 (−2.88 to 5.52)	0.06	0.54
Baseline $KOOS_4$	0.22 (0.09 to 0.36)	0.30	**0.001**
Age	−0.31 (−0.59 to −0.03)	−0.21	**0.03**
Male gender	−1.73 (−7.44 to 3.98)	−0.07	0.55
Contact pivoting sports participation	1.67 (−3.27 to 6.61)	0.07	0.50
Graft diameter[d]	3.08 (−0.74 to 6.90)	0.16	0.11
Partial meniscectomy	4.03 (−0.65 to 8.71)	0.15	0.09

Abbreviations: KOOS, Knee Injury and Osteoarthritis Outcome Score; PRP, platelet-rich plasma.

a. The $KOOS_4$ is calculated as the average score for 4 subscales (pain, symptoms, sports and recreation, and quality of life) of the KOOS; scores range from 0 to 100, with higher scores indicating better knee-related results (patient-acceptable symptom state threshold, 70.9).

b. The enter method is applied to determine the independent predictors. Of the 7 included variables, group allocation (PRP group vs control group), gender (male vs female), sports participation (contact pivoting vs non-contact-pivoting), and meniscal treatment (partial medial or lateral meniscectomy vs none) are inputted as dichotomous variables (1 vs 0, respectively), while baseline $KOOS_4$, age, and graft diameter are continuous variables.

c. The regression coefficients B and beta (standardized) represent the effect of an independent variable on the outcome ($KOOS_4$), with positive or negative values indicating improved outcome scores resulting from increased or decreased values of the variable, respectively.

d. The graft diameter is calculated as the quadratic mean of the graft diameters of the anteromedial and posterolateral bundles and inputted as an independent variable to avoid the multicollinearity caused by 2 strongly-correlated variables.

© 2024 Ye Z et al. *JAMA Network Open*.

eTable 4. Physical Examinations During Follow-Up Visits (Primary Analysis)

Outcome	PRP group	Control group	*P* value
Range of motion[a] (flexion), deg			
4 wk, mean (SD)	92 (17)	88 (18)	0.29
8 wk, mean (SD)	110 (14)	107 (16)	0.33
3 mo, mean (SD)	120 (11)	117 (14)	0.16
6 mo, median (IQR)	130 (121 to 135)	130 (120 to 135)	0.39
12 mo, median (IQR)	135 (130 to 140)	135 (125 to 140)	0.21
Range of motion (extension), deg			
4 wk, median (IQR)	3 (0 to 5)	5 (0 to 5)	0.77
8 wk, median (IQR)	0 (0 to 5)	0 (0 to 5)	0.82
3 mo, median (IQR)	0 (−3 to 0)	0 (0 to 3)	0.16
6 mo, median (IQR)	−2 (−3 to 0)	0 (−2 to 0)	0.09
12 mo, median (IQR)	−3 (−5 to 0)	−2 (−5 to 0)	0.30
Knee circumference[b], SSD, mm			
4 wk, median (IQR)	2 (1 to 2)	1 (1 to 2)	0.51
8 wk, median (IQR)	1 (0 to 2)	1 (1 to 2)	0.83
3 mo, median (IQR)	1 (0 to 1)	1 (1 to 1)	0.42
6 mo, median (IQR)	0 (0 to 1)	1 (0 to 1)	0.22
12 mo, median (IQR)	0 (0 to 0)	0 (0 to 0)	0.33
Grades of knee laxity at 12 mo[c]			
Anterior drawer test, 0/1/2/3, No.	54/3/0/0	55/2/0/0	0.65
Lachman test, 0/1/2/3, No.	54/0/3/0	55/0/0/2	0.68
Pivot shift test, 0/1/2/3, No.	54/2/1/0	55/0/1/1	0.68
KT-1000, SSD, median (IQR), mm	0 (0 to 2)	0 (0 to 2)	0.43

Abbreviations: IQR, interquartile range; PRP, platelet-rich plasma; SSD, side-to-side difference.

a. The active-assisted range of motion is measured by placing the axis of a goniometer over the lateral femoral epicondyle and lining the two arms along the femoral and fibular axes, with positive values indicating knee flexion and negative values indicating hyperextension. The knee flexion and extension are assisted by both arms of the participant (in the sitting position) and a firm pillow beneath the heel (in the supine position), respectively.

b. The knee circumference is measured by placing a tape measure circumferentially around the knee at the mid-patellar level while the participant is in the supine position with full knee extension. The result is presented as the SSD by subtracting the circumference of the contralateral knee, with higher positive values indicating worse swelling.

c. The anterior drawer and Lachman tests represent knee anteroposterior laxity at 90° and 30° of flexion, respectively; grades range from 0 (0−2 mm) to 3 (>10 mm) based on the SSD. The pivot-shift test is graded as 0 (negative), 1 (glide), 2 (clunk), and 3 (gross reduction), with higher grades indicating increased knee rotatory laxity. The KT-1000 arthrometer is a

© 2024 Ye Z et al. *JAMA Network Open*.

widely-used device for quantitative measurements of the anterior tibial translation at 30° of knee flexion, with higher SSD values indicating increased knee anteroposterior laxity.

© 2024 Ye Z et al. *JAMA Network Open*.

eTable 5. Patient-Reported Outcomes During Follow-Up Visits (Per-Protocol Analysis)[a]

Outcome	PRP group	Control group	Adjusted between-group difference[b] (95% CI)	*P* value
Primary outcome				
No. of participants	53	54	—	—
$KOOS_4$[c] at 12 mo, mean (SD)	79.4 (11.5)	76.9 (11.8)	2.7 (−1.6 to 7.0)	0.22
Secondary outcomes				
GROC[d] at 12 mo, median (IQR)	+5 (+4 to +6)	+5 (+3 to +5)	—	0.42
PROs at 3 mo, mean (SD)				
No. of participants	55	55	—	—
KOOS pain	77.5 (14.4)	79.3 (16.2)	−1.1 (−6.8 to 4.6)	0.70
KOOS symptoms	61.6 (16.4)	61.2 (19.0)	0.8 (−5.8 to 7.3)	0.82
KOOS activities of daily living	87.4 (12.0)	88.1 (11.4)	−0.7 (−5.2 to 3.7)	0.74
KOOS sports and recreation	48.4 (26.2)	50.1 (25.8)	−0.9 (−10.2 to 8.4)	0.85
KOOS quality of life	41.8 (20.1)	45.0 (22.2)	−3.4 (−10.7 to 3.9)	0.36
P_4 score[e] by VAS	6.5 (5.4)	6.3 (4.8)	0 (−1.9 to 1.8)	0.96
Tegner score[f]	2.5 (1.1)	2.5 (1.3)	0 (−0.4 to 0.5)	0.90
Lysholm score[g]	71.2 (17.3)	73.6 (17.3)	−2.3 (−8.6 to 4.0)	0.47
Subjective IKDC score[h]	60.0 (14.2)	61.0 (15.3)	−1.1 (−6.6 to 4.3)	0.68
PROs at 6 mo, mean (SD)				
No. of participants	55	54	—	—
KOOS pain	86.6 (10.6)	84.0 (10.6)	3.2 (−0.6 to 7.1)	0.10
KOOS symptoms	74.0 (16.2)	69.0 (18.8)	5.4 (−1.2 to 11.9)	0.11
KOOS activities of daily living	95.0 (6.6)	92.4 (9.2)	2.6 (−0.4 to 5.6)	0.09
KOOS sports and recreation	74.4 (18.1)	68.2 (17.8)	6.9 (0.5 to 13.3)	**0.04**
KOOS quality of life	56.8 (20.0)	51.2 (21.0)	5.7 (−1.9 to 13.2)	0.14
P_4 score by VAS	2.8 (2.7)	3.2 (3.4)	−0.5 (−1.6 to 0.7)	0.42
Tegner score	4.5 (1.7)	3.7 (1.3)	0.8 (0.3 to 1.3)	**0.002**
Lysholm score	83.0 (13.0)	80.2 (13.6)	2.9 (−1.9 to 7.7)	0.23
Subjective IKDC score	75.4 (11.0)	73.0 (11.3)	2.2 (−1.8 to 6.2)	0.27
PROs at 12 mo, mean (SD)				
No. of participants	53	54	—	—
KOOS pain	89.3 (9.0)	87.5 (9.2)	2.1 (−1.2 to 5.5)	0.21
KOOS symptoms	77.4 (13.5)	77.1 (15.0)	0.3 (−4.9 to 5.6)	0.90
KOOS activities of daily living	96.7 (4.8)	94.9 (7.1)	1.7 (−0.6 to 4.1)	0.14
KOOS sports and recreation	82.4 (15.5)	80.2 (15.4)	2.5 (−3.3 to 8.2)	0.40
KOOS quality of life	68.4 (18.6)	62.6 (18.2)	5.8 (−1.2 to 12.8)	0.10
P_4 score by VAS	1.8 (2.2)	2.0 (2.7)	−0.3 (−1.3 to 0.6)	0.51
Tegner score	5.6 (1.9)	5.4 (1.6)	0.3 (−0.2 to 0.8)	0.27
Lysholm score	84.6 (11.6)	83.8 (11.4)	0.7 (−3.6 to 5.1)	0.74
Subjective IKDC score	82.0 (11.2)	79.9 (9.8)	1.8 (−2.1 to 5.6)	0.36

© 2024 Ye Z et al. *JAMA Network Open.*

Abbreviations: GROC, Global Rating of Change; IKDC, International Knee Documentation Committee; IQR, interquartile range; KOOS, Knee Injury and Osteoarthritis Outcome Score; PRO, patient-reported outcome; PRP, platelet-rich plasma; VAS, visual analog scale.

a. The per-protocol analysis includes 55 participants in the PRP group and 57 participants in the control group. The PRP group excludes 5 participants: 2 received 1 dose of PRP injection (unwilling to continue because of knee swelling and pain, as reported in adverse events), 1 received 2 doses of PRP injection (unwilling to continue because of time consumption), and 2 received reoperation (1 for arthrolysis because of limited range of motion and 1 for meniscal repair because of reinjury). The control group excludes 3 participants receiving reoperation (for arthrolysis because of limited range of motion).

b. The mean between-group difference for each subjective outcome (except for GROC scale) is adjusted for the corresponding baseline (preoperative) score using an analysis of covariance model, with positive values indicating higher scores in the PRP group and negative values indicating higher scores in the control group.

c. KOOS includes 42 items covering 5 subscales (pain, symptoms, activities of daily living, sports and recreation, and quality of life); scores for each subscale range from 0 to 100, with higher scores indicating better knee-related results. The $KOOS_4$ is calculated as the average score for 4 of the 5 subscales (except for activities of daily living).

d. GROC scale assesses the overall change of condition resulting from the treatment (surgery and postoperative treatment); scores range from −7 (a very great deal worse) to +7 (a very great deal better), with higher positive values indicating more improvement.

e. The VAS for pain is assessed on a straight horizontal line, with scores ranging from 0 (no pain) to 10 (worst pain possible). The P_4 score is calculated as the sum of VAS scores at 4 timepoints (morning, afternoon, evening, and with activity) during the past 2 days.

f. Tegner Activity Scale assesses the highest level of current sports participation; scores range from 0 (sick leave or disability) to 10 (professional level of competitive sports).

g. Lysholm Knee Questionnaire includes 8 items regarding subjective perception (such as instability, pain, and locking) related to knee ligament injury; scores range from 0 to 100, with higher scores indicating fewer symptoms and better function in daily living.

h. IKDC Subjective Knee Form includes 18 items covering 3 domains (symptoms, sports activities, and function); scores range from 0 (worst condition) to 100 (best condition).

© 2024 Ye Z et al. *JAMA Network Open*.

eTable 6. Graft Maturity on MRI During Follow-Up Visits (Per-Protocol Analysis)[a]

Outcome	PRP group	Control group	*P* value
Graft SNQ[b] at 3 mo, median (IQR)			
No. of participants	55	55	—
Anteromedial bundle			
Femoral intra-tunnel segment[c]	10.0 (5.7 to 15.8)	11.0 (4.4 to 16.5)	0.71
Intra-articular segment	6.7 (4.2 to 11.6)	6.1 (3.0 to 9.9)	0.37
Tibial intra-tunnel segment	6.1 (2.4 to 9.7)	4.4 (2.1 to 7.5)	0.15
Posterolateral bundle			
Femoral intra-tunnel segment	9.8 (4.4 to 15.1)	11.3 (5.2 to 17.5)	0.53
Intra-articular segment	8.7 (5.3 to 13.0)	7.8 (3.8 to 12.4)	0.36
Tibial intra-tunnel segment	6.0 (4.0 to 9.8)	5.2 (2.8 to 8.3)	0.42
Graft SNQ at 6 mo, median (IQR)			
No. of participants	55	54	—
Anteromedial bundle			
Femoral intra-tunnel segment	13.4 (10.2 to 17.7)	17.5 (12.3 to 22.6)	**0.003**
Intra-articular segment	10.0 (5.4 to 13.8)	13.0 (8.6 to 17.4)	**0.04**
Tibial intra-tunnel segment	10.2 (6.5 to 14.2)	9.8 (5.3 to 15.3)	0.76
Posterolateral bundle			
Femoral intra-tunnel segment	15.4 (11.6 to 21.0)	19.7 (14.2 to 24.2)	**0.02**
Intra-articular segment	11.1 (6.4 to 17.4)	14.9 (11.1 to 18.9)	**0.01**
Tibial intra-tunnel segment	11.9 (6.1 to 17.2)	10.1 (5.8 to 16.8)	0.71
Graft SNQ at 12 mo, median (IQR)			
No. of participants	53	54	—
Anteromedial bundle			
Femoral intra-tunnel segment	9.8 (6.1 to 17.4)	12.6 (8.1 to 17.5)	0.11
Intra-articular segment	8.1 (5.6 to 11.4)	9.6 (5.3 to 14.2)	0.29
Tibial intra-tunnel segment	8.3 (5.9 to 11.1)	8.2 (4.9 to 11.8)	0.82
Posterolateral bundle			
Femoral intra-tunnel segment	10.4 (6.2 to 19.3)	13.2 (8.9 to 20.3)	0.17
Intra-articular segment	7.9 (5.5 to 11.4)	10.7 (5.9 to 15.0)	0.08
Tibial intra-tunnel segment	7.2 (5.1 to 10.8)	7.2 (4.0 to 11.4)	0.81

Abbreviations: IQR, interquartile range; MRI, magnetic resonance imaging; PRP, platelet-rich plasma; SNQ, signal-to-noise quotient.

a. The per-protocol analysis includes 55 participants in the PRP group and 57 participants in the control group. The PRP group excludes 5 participants: 2 received 1 dose of PRP injection (unwilling to continue because of knee swelling and pain, as reported in adverse events), 1 received 2 doses of PRP injection (unwilling to continue because of time consumption), and 2 received reoperation (1 for arthrolysis because of limited range of

© 2024 Ye Z et al. *JAMA Network Open.*

motion and 1 for meniscal repair because of reinjury). The control group excludes 3 participants receiving reoperation (for arthrolysis because of limited range of motion).

b. The graft maturity is evaluated on the sagittal plane of the fat-saturated proton-density-weighted MRI. The SNQ is calculated as: (signal intensity of ACL graft – signal intensity of quadriceps tendon) / signal intensity of background, with lower SNQ values indicating better graft maturity. The quadriceps tendon and background are located at the patellar upper limit level and at 2 cm anterior to the patellar tendon, respectively.

c. The SNQ values are separately calculated for 6 segments of the reconstructed ACL: the femoral intra-tunnel segment, intra-articular segment, and tibial intra-tunnel segment of the anteromedial bundle and posterolateral bundle, respectively. In each segment, 3 regions of interest (0.1-cm^2 circles) are selected, and the mean signal intensity is calculated.

© 2024 Ye Z et al. *JAMA Network Open*.

eTable 7. Physical Examinations During Follow-Up Visits (Per-Protocol Analysis)[a]

Outcome	PRP group	Control group	*P* value
Range of motion[b] (flexion), deg			
4 wk, mean (SD)	91 (18)	90 (17)	0.71
8 wk, mean (SD)	110 (15)	108 (14)	0.61
3 mo, mean (SD)	121 (11)	119 (12)	0.32
6 mo, median (IQR)	131 (122 to 135)	130 (124 to 135)	0.40
12 mo, median (IQR)	135 (130 to 140)	135 (125 to 140)	0.27
Range of motion (extension), deg			
4 wk, median (IQR)	3 (0 to 5)	5 (0 to 5)	0.93
8 wk, median (IQR)	0 (0 to 5)	0 (0 to 5)	0.87
3 mo, median (IQR)	0 (−3 to 0)	0 (0 to 3)	0.23
6 mo, median (IQR)	−2 (−3 to 0)	0 (−2 to 0)	0.17
12 mo, median (IQR)	−3 (−5 to 0)	−2 (−5 to 0)	0.32
Knee circumference[c], SSD, mm			
4 wk, median (IQR)	2 (1 to 2)	1 (1 to 2)	0.68
8 wk, median (IQR)	1 (0 to 2)	1 (1 to 2)	0.89
3 mo, median (IQR)	1 (0 to 1)	1 (1 to 1)	0.70
6 mo, median (IQR)	0 (0 to 1)	1 (0 to 1)	0.36
12 mo, median (IQR)	0 (0 to 0)	0 (0 to 0)	0.47
Grades of knee laxity at 12 mo[d]			
Anterior drawer test, 0/1/2/3, No.	51/2/0/0	52/2/0/0	0.65
Lachman test, 0/1/2/3, No.	51/0/2/0	52/0/0/2	0.68
Pivot shift test, 0/1/2/3, No.	51/2/0/0	52/0/1/1	0.68
KT-1000, SSD, median (IQR), mm	1 (0 to 2)	0 (0 to 2)	0.57

Abbreviations: IQR, interquartile range; PRP, platelet-rich plasma; SSD, side-to-side difference.

a. The per-protocol analysis includes 55 participants in the PRP group and 57 participants in the control group. The PRP group excludes 5 participants: 2 received 1 dose of PRP injection (unwilling to continue because of knee swelling and pain, as reported in adverse events), 1 received 2 doses of PRP injection (unwilling to continue because of time consumption), and 2 received reoperation (1 for arthrolysis because of limited range of motion and 1 for meniscal repair because of reinjury). The control group excludes 3 participants receiving reoperation (for arthrolysis because of limited range of motion).

b. The active-assisted range of motion is measured by placing the axis of a goniometer over the lateral femoral epicondyle and lining the two arms along the femoral and fibular axes, with positive values indicating knee flexion and negative values indicating hyperextension. The knee flexion and extension are assisted by both arms of the participant (in the sitting position) and a firm pillow beneath the heel (in the supine position), respectively.

© 2024 Ye Z et al. *JAMA Network Open*.

c. The knee circumference is measured by placing a tape measure circumferentially around the knee at the mid-patellar level while the participant is in the supine position with full knee extension. The result is presented as the SSD by subtracting the circumference of the contralateral knee, with higher positive values indicating worse swelling.

d. The anterior drawer and Lachman tests represent knee anteroposterior laxity at 90° and 30° of flexion, respectively; grades range from 0 (0−2 mm) to 3 (>10 mm) based on the SSD. The pivot-shift test is graded as 0 (negative), 1 (glide), 2 (clunk), and 3 (gross reduction), with higher grades indicating increased knee rotatory laxity. The KT-1000 arthrometer is a widely-used device for quantitative measurements of the anterior tibial translation at 30° of knee flexion, with higher SSD values indicating increased knee anteroposterior laxity.

© 2024 Ye Z et al. *JAMA Network Open*.

eTable 8. Medications and Adverse Events During Follow-Up Visits

Event	PRP group	Control group	*P* value[a]
Pain-relieving medication use[b], No. (%)			
Acetaminophen at 3 mo	15 (25.4)	13 (22.4)	0.70
NSAIDs at 3 mo	2 (3.4)	4 (6.9)	0.44
Acetaminophen at 6 mo	6 (10.2)	5 (8.8)	0.80
NSAIDs at 6 mo	1 (1.7)	3 (5.3)	0.36
Acetaminophen at 12 mo	0 (0)	3 (5.3)	0.24
NSAIDs at 12 mo	0 (0)	0 (0)	—
Unscheduled knee injection, No. (%)	0 (0)	0 (0)	—
Intervention-related adverse events[c]			
Fainting, No. (%)	0 (0)	—	—
Allergic reaction, No. (%)	0 (0)	—	—
Pain at injection site, No. (%)	4 (6.7)	—	—
Duration of symptom, median (range), d	2 (1 to 3)	—	—
Knee swelling, No. (%)	3 (5.0)	—	—
Duration of symptom, median (range), d	3 (1 to 5)	—	—
Decreased range of motion, No. (%)	0 (0)	—	—
Infection at injection site, No. (%)	0 (0)	—	—
Common adverse events[d], No. (%)			
Graft rupture	0 (0)	0 (0)	—
Residual knee laxity[e]	3 (5.0)	2 (3.3)	>0.99
Limited range of motion[f]	3 (5.0)	5 (8.3)	0.72
Reoperation[g]	2 (3.3)	3 (5.0)	>0.99

Abbreviations: NSAID, non-steroidal anti-inflammatory drug; PRP, platelet-rich plasma.

a. The between-group difference is analyzed using the Pearson chi-square test or Fisher exact test depending on expected frequencies for the 2×2 contingency table.

b. The pain-relieving medications during the past 2 weeks were inquired at each follow-up visit to assess the risk of potential confounding biases. Acetaminophen was usually prescribed for participants with a visual analog scale >3 of 10 if required. Prescription of NSAIDs (such as ibuprofen and celecoxib) was avoided because of the potential to hamper tendon-to-bone healing processes, while several participants reported self-administration.

c. The intervention-related adverse events were reported by 5 participants: 2 reported knee swelling and pain (1 relieved in 5 days; 1 relieved in 3 days) at injection dose 1 and were unwilling to receive subsequent doses, 1 reported knee swelling at dose 1 (relieved in 1 day), and 2 reported pain at injection site (1 at dose 2, and relieved in 1 day; 1 at dose 3, and relieved in 2 days). No serious intervention-related adverse events were observed.

d. The common adverse events have been observed and treated in patients undergoing anterior cruciate ligament reconstruction (ACLR) and may not be associated with PRP injection.

© 2024 Ye Z et al. *JAMA Network Open*.

e. Residual knee laxity includes excessive anterior tibial translation (anterior drawer test or Lachman test grade ≥2 of 3, or KT-1000 side-to-side difference >5 mm) and residual pivot shift (pivot-shift test grade 2 or 3, or a persistent pivot-shift test grade 1).

f. Limited range of motion (after ACLR) includes knee flexion <90° or knee extension >15° at 3 months and knee flexion <120° or knee extension >5° at 12 months.

g. Reoperation was performed based on shared decision-making for 5 participants: 4 (1 in the PRP group and 3 in the control group) received arthrolysis because of limited range of motion and 1 (in the PRP group) received meniscal repair because of reinjury.

© 2024 Ye Z et al. *JAMA Network Open*.
