# Supplementary material for: Intra-Articular Platelet-Rich Plasma Injection After Anterior Cruciate Ligament Reconstruction: A Randomized Clinical Trial
Source: JAMA Netw Open. 2024 May 10;7(5):e2410134. doi: 10.1001/jamanetworkopen.2024.10134 (PMC11087838; doi:10.1001/jamanetworkopen.2024.10134)
Supplement: Supplement 3. — Data Sharing Statement [file jamanetwopen-e2410134-s003.pdf]

Data Sharing Statement

Ye. Intra-Articular Platelet-Rich Plasma Injection After Anterior Cruciate Ligament Reconstruction. *JAMA Netw Open*. Published May 10, 2024.
doi:10.1001/jamanetworkopen.2024.10134

Data
Data available: Yes
Data types: Data (not involving human participants)
How to access data: Researchers may send an e-mail to jzzhao@sjtu.edu.cn for data inquiry
When available: With publication

Supporting Documents
Document types: None

Additional Information
Who can access the data: Researchers whose proposed use of the data has been approved
Types of analyses: For research use such as systematic reviews and meta-analyses
Mechanisms of data availability: After approval of a proposal and with a signed data access agreement
